# Generation and applications of simulated datasets to integrate social network and demographic analyses

**DOI:** 10.1002/ece3.9871

**Published:** 2023-05-15

**Authors:** Matthew J. Silk, Olivier Gimenez

**Affiliations:** ^1^ CEFE, Univ Montpellier, CNRS, EPHE, IRD Montpellier France

**Keywords:** co‐capture data, hidden Markov model, population dynamics, stochastic block model, survival

## Abstract

Social networks are tied to population dynamics; interactions are driven by population density and demographic structure, while social relationships can be key determinants of survival and reproductive success. However, difficulties integrating models used in demography and network analysis have limited research at this interface. We introduce the R package genNetDem for simulating integrated network–demographic datasets. It can be used to create longitudinal social network and/or capture–recapture datasets with known properties. It incorporates the ability to generate populations and their social networks, generate grouping events using these networks, simulate social network effects on individual survival, and flexibly sample these longitudinal datasets of social associations. By generating co‐capture data with known statistical relationships, it provides functionality for methodological research. We demonstrate its use with case studies testing how imputation and sampling design influence the success of adding network traits to conventional Cormack–Jolly–Seber (CJS) models. We show that incorporating social network effects into CJS models generates qualitatively accurate results, but with downward‐biased parameter estimates when network position influences survival. Biases are greater when fewer interactions are sampled or fewer individuals observed in each interaction. While our results indicate the potential of incorporating social effects within demographic models, they show that imputing missing network measures alone is insufficient to accurately estimate social effects on survival, pointing to the importance of incorporating network imputation approaches. genNetDem provides a flexible tool to aid these methodological advancements and help researchers testing other sampling considerations in social network studies.

## Graphical abstract

Difficulties integrating models used in demography and network analysis have limited research at the interface of social and population ecology. Here, we introduce the R package genNetDem for simulating integrated network–demographic datasets for methodological research. We demonstrate its use with case studies testing how imputation and sampling design influence our ability to estimate social network effects on survival in capture–recapture data.

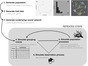

## 1 INTRODUCTION

Network analysis has revolutionized animal social behavior research by quantifying how dyadic social interactions and relationships are nested in wider group‐ and population‐level social structures (Krause et al., 2014; Pinter‐Wollman et al., 2013). Network studies in behavioral ecology have often focused on how the position of an individual within its social network influences its fitness, either via reproductive success (Formica et al., 2012; Oh & Badyaev, 2010) or survival (Blumstein et al., 2018; Ellis et al., 2017; Stanton & Mann, 2012).

Quantifying direct links between social network position and fitness can help us understand how selection acts on social behavioral traits. Furthermore, determining how social behavior is linked to survival can identify demographic consequences of interactions and associations (Clements et al., 2022), which can help develop more realistic models for how social species respond to population declines or environmental change (Snijders et al., 2017). However, while there is growing interest in linking animal social networks with demography (Shizuka & Johnson, 2020), there remain many methodological challenges.

Currently, most studies that link network position and fitness use known fate approaches such as generalized linear models (e.g., Blumstein et al., 2018) or Cox proportional‐hazards models (e.g., Ellis et al., 2017). However, application of these approaches is limited in many wild populations where individuals that are alive are not necessarily detected. In these cases, survival is most commonly estimated using hidden Markov models (HMMs; McClintock et al., 2020) that can simultaneously estimate survival and probabilities of capture (Gimenez et al., 2012; Pradel, 2005). These models also have potential as tools in animal social network analysis (Clements et al., 2022; Fisher et al., 2017), especially when not all associations are detected. However, it is challenging to provide universal guidance on the applicability of these approaches given the diversity of animal social systems and sampling designs used to study them.

Here, we introduce the R package genNetDem to simulate co‐capture datasets. We define a co‐capture dataset as one in which capture–recapture data also provide information on social structure, such as when individuals are caught or observed in groups (see also Silk et al., 2021). The package generates integrated longitudinal social network and capture–recapture datasets with known statistical relationships. This provides functionality for methodological research, power analyses, and sampling design. Here, we present an overview the package, outline effective workflows and describe key functions. We then provide two case studies to demonstrate its use. Finally, we identify key next steps in merging social network and demographic analyses, and discuss the role of genNetDem in these.

## 2 genNetDem OVERVIEW

**FIGURE 1:**
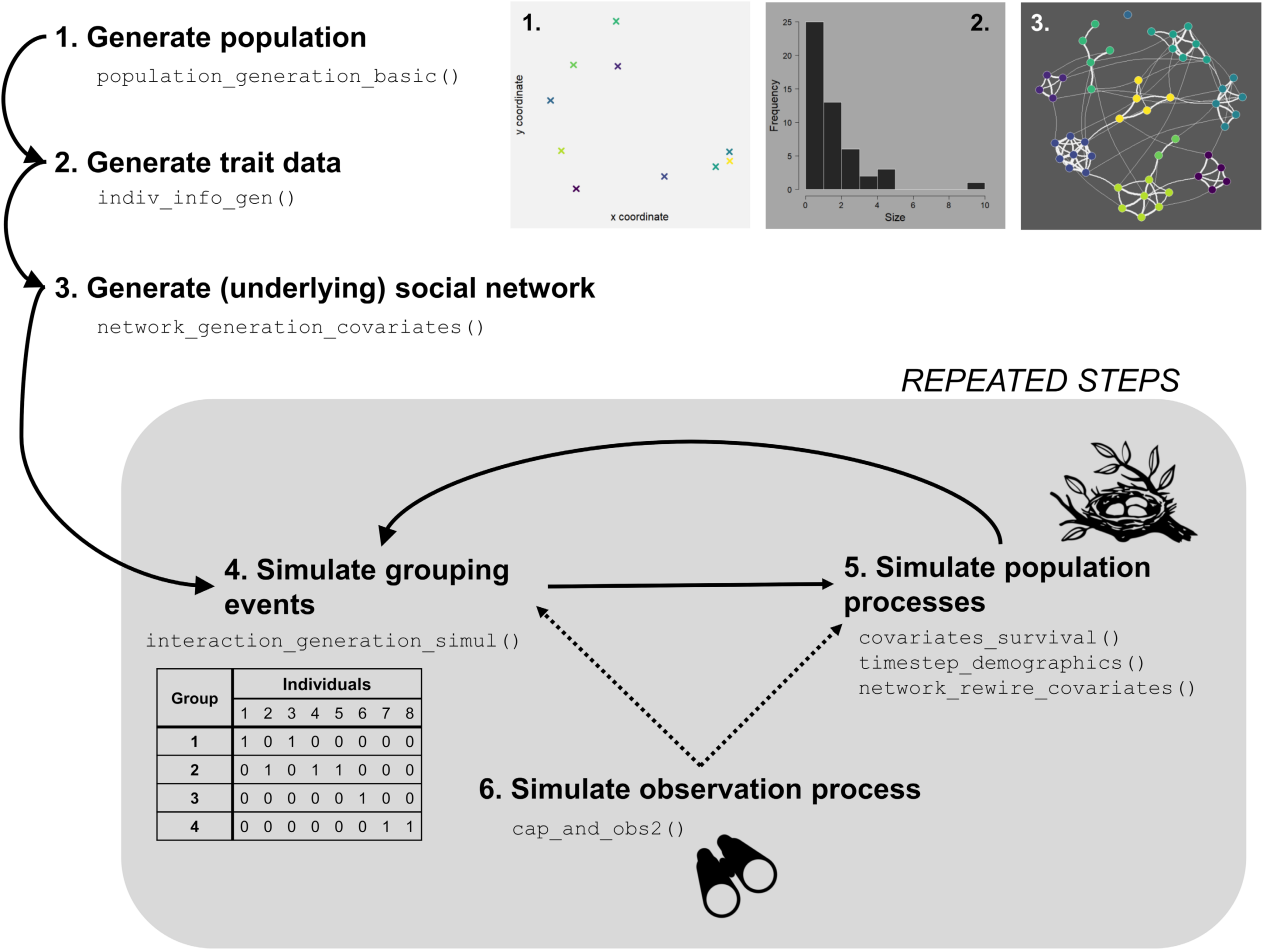
An example workflow for using genNetDem to simulate integrated network‐demographic datasets. This is a simplified version of the approach used in the case studies with the gray box capturing a demographic timestep and step 4 involving one or more behavioral timesteps. Note that while in our case studies, we fix a relationship between social network position and survival prior to the repeated steps, this relationship in the covariates_survival() function could vary between timesteps if desired, hence its inclusion within the repeated steps box. Note also that multiple set of interactions can be generated prior to the simulation of population processes. The modular nature of the functions in the package means that different parts of this workflow can also be used independently. Further usage examples are provided in the package vignettes.

genNetDem is a set of R (R Core Team, 2021) functions that generate longitudinal social network and/or capture–recapture datasets with known underlying properties. Functionality can be split into four broad groups: (a) population features; (b) survival features; (c) social network features; and (d) observation features. The package is modular meaning‐specific components can be used in isolation or user‐generated code can be integrated to extend functionality to different ecological or social contexts. Here, we provide an idea of potential workflows when using genNetDem including a detailed example (Figure 1) and an overview of key functions (Table 1; with more detail provided in the Appendices S1 and S2). genNetDem is available on GitHub (https://github.com/NETDEM‐project/genNetDem).

**TABLE 1:** An overview of the main functions provided by genNetDem alongside information on their main inputs and outputs. Note that where multiple similar functions exist, we include one example in the table, but alternatives are detailed in the main text and Appendix S2.

Function	Purpose	Main inputs	Main outputs
population_generation_basic()	Generates initial population	Number of individuals Number of groups	Population dataframe Distance matrix
indiv_info_gen()	Generates individual trait data	Population dataframe Trait information	Trait dataframe
indiv_info_add()	Adds individual trait data for recruited individuals	Population dataframe Trait dataframe	Updated trait dataframe
timestep_demographics()	Controls survival and recruitment at the end of each demographic timestep	Population dataframe	Updated population dataframe Updated distance matrix
covariates_survival()	Simulates survival probabilities for each individual based on its network position and individual traits	Population dataframe Trait dataframe Network Effect sizes	Updated population dataframe
network_generation_covariates()	Simulates the (underlying) social network structure of the population	Population dataframe Trait dataframe Distance matrix Generative model parameters	Social network as an adjacency matrix and igraph object
network_rewire_covariates()	Generates social network for newly recruited individuals and allows rewiring of social connections for existing individuals	Population dataframe Previous population dataframe Trait dataframe Distance matrix Generative model parameters	Updated social network (as an adjacency matrix and igraph object)
interaction_generation_simul()	Generates association data (grouping events) based on the population social network	Population dataframe Social network Mean group size	Group‐by‐individual matrix (incidence matrix) linking individuals to particular grouping events
network_checker_simul()	Compares properties of the network generated from grouping events to underlying social network	Group‐by‐individual matrix Social network	Results of the comparisons carried out
cap_and_obs2()	Simulates observation process in each behavioral timestep by imperfect sampling of grouping events	Group‐by‐individual matrix Sampling parameters	Group‐by‐individual matrices for captured and observed groups
cap_dat_gen()	Converts captures and observations into conventional capture‐recapture datasets	Overall population dataframe Information on sampling	Capture‐recapture data for demographic and behavioral timesteps

## 3 genNetDem WORKFLOW

While genNetDem is designed to be modular so that individual components can be adjusted to perform a range of tasks, many of the functions fit well within specific workflows. We illustrate one such common workflow (Figure 1), but various other applications are demonstrated in package vignettes. The workflow illustrated here generates a population with a known, underlying social structure and then simulates grouping events (or associations) using this underlying social structure alongside demographic change, sampling from the grouping events to simulate an observation process.

1. *Population generation:*
  genNetDem provides functionality to simulate a population of a given size that can be subdivided into a prespecified number of (underlying) social groups distributed in 2D space.
2. *Generation of trait data:*
  genNetDem can also be used to simulate trait data for individuals in the population with considerable flexibility in the types of traits that could be included. It is also possible to use existing biological data or external methods of simulating trait data if preferred as long as the datasets are then formatted in an equivalent manner.
3. *Generate social network:*
  A key feature of genNetDem is a generative model of underlying social network structures that uses provided information on the presence of social groups, the spatial structure of the population, and traits of individuals within it using an adapted stochastic block model (Lee & Wilkinson, 2019). We use ***social group*** to refer to the assignment of individuals to prespecified groups when populations are generated, and ***spatial structure*** as any additional effects attributed to the distribution of these groups in 2D space. While using this inbuilt functionality is appealing due to the required inputs and outputs being adapted for other parts of the package, it is equally possible to use other tools to simulate the underlying social network structure. For example, users may want to employ standard generative models (e.g., erdos‐renyi random graphs and small‐world networks) or to take advantage of the growing availability of more advanced and highly flexible generative models for networks. One example is the STRAND R package (Ross et al., 2022) that combines features from the social relations model alongside the stochastic block model.
4. *Simulate interactions:*
  It is then possible to use genNetDem to simulate social interactions using this underlying social structure. These interactions/events can incorporate dyadic or nondyadic interactions; hence, our use of grouping events to describe these (higher‐order) interactions generated from an underlying dyadic network of social relationships.
5. *Simulate population processes:*
  genNetDem additionally provides functionality to simulate survival and recruitment to incorporate population dynamics. Survival can be simulated as a function of an individual's social interactions and nonsocial traits enabling genNetDem to provide a powerful tool to better understand links between social behavior and population processes. Currently, recruitment is strongly density‐dependent as a tool to maintain an (approximately) constant population size.
6. *Simulate an observation process:*
  Finally, genNetDem also provides tools to simulate a capture and observation process based on the simulated grouping events (interactions) such that it is inherently influenced by the underlying social structure. These samples can be used to generate co‐capture datasets to test the power to detect social network effects on demographic rates (as illustrated in the case studies) or other research questions of interest.

In a typical workflow, these stages can be linked together to generate longitudinal datasets. For example, in Figure 1, we repeat steps 4, 5, and 6 to generate a co‐capture dataset that provides a window into how social network structure and demographic rates are linked in our simulated population. Adapted versions of this workflow are used for the case studies below.

## 4 genNetDem FUNCTIONS

We provide a description of key functions here and summary in Table 1, with more technical details on functions provided in the Appendix S2.

### 4.1 Population features

The population features provide capability to simulate a population and generate data about individuals in it. There is then functionality that simulates population dynamics based on individual survival probabilities (see Section 4.2) and stochastic recruitment that maintains an approximately stable population size when employed.

The **population_generation_basic()** function generates data for a group‐structured population distributed uniformly in 2D space. The function takes two arguments: *n* defines the population size and *ng* the number of groups in the population. When *n* = *ng,* individuals are distributed uniformly at random across the defined coordinates. When *n* > *ng,* groups are distributed uniformly at random across the same coordinates with individuals in the same group sharing the same spatial location. Currently, simulated population size is independent of the extent of the area it occupies. Therefore, population density will increase with population size and impact spatial effects on social network structure. This does not represent a problem except when users want to compare the social structures of populations of different sizes. Group membership is currently fixed once an individual is recruited into the population (although future versions are likely to allow more flexibility in group membership). The **indiv_info_gen()** and **indiv_info_add()** functions provide flexibility in generating and updating individual‐level trait data. Variables can be specified as covariates (e.g., size) or categorical factors (e.g., sex), with further arguments specifying additional features of the variable (e.g., the distribution of a covariate or the number of levels and level names of a factor). Trait values are assigned stochastically using the indiv_info_gen() function, but it is also possible to use researcher‐defined trait values if they are formatted in an appropriate manner for the package.

The **timestep_demographics()** function controls survival and recruitment in the simulated population. Survival is stochastic based on each individual's survival probability (see Section 4.2). The number of recruits is Poisson distributed ($\lambda ={\bar{\mathrm{Pr}\left(\text{Survival}\right)}}^{-1}-1$, where $\bar{\mathrm{Pr}\left(\text{Survival}\right)}\;$ is the mean survival probability in the population) to be approximately density‐dependent. When the population is group‐structured, individuals can only be recruited into existing groups. When there is no underlying group structure, then individuals are recruited into existing locations if they are available and new locations otherwise.

### 4.2 Survival features

The **covariates_survival()** function allows survival probabilities to be calculated for each individual based on individual traits and the position of an individual within a population social network (this could be any network provided to the function; the underlying social networks, simulated interaction network or a separate user‐specified network). Individual traits specified in the dataframe generated by indiv_info_gen() can be used as covariates. There is also considerable flexibility in which measures of network position can be included as covariates; both the function and R package used can be specified within the function, with functionality for most common packages (e.g., sna: Butts, 2014; igraph: Csardi & Nepusz, 2006; tnet: Opsahl, 2009) incorporated. It is also possible to simulate network covariance in survival whereby closely connected individuals have either more or less similar survival probabilities than expected by chance (this uses an approximation of the underlying network that is positive definite as a covariance matrix). Note that it may also be possible for some network covariance in survival probabilities to arise without this being encoded directly, for example if survival is positively associated with centrality and more central individuals tend to be more connected with each other. Currently, covariates_survival() simulates independent (additive) effects of traits, meaning that, while the effects of multiple traits can be incorporated together, there is no functionality to capture interactions among variables (e.g., network position having different effects in males than females). The simpler **basic_survival()** function generates population‐level survival probabilities in the absence of covariates.

### 4.3 Network features

There are two core functionalities of the network features: to generate underlying social networks for the population and to generate grouping events (interactions/associations) based on these networks. There are also two ***network_checker()*** functions that quantify and visualize how well social networks derived from grouping events match the underlying network used to generate them.

**FIGURE 2:**
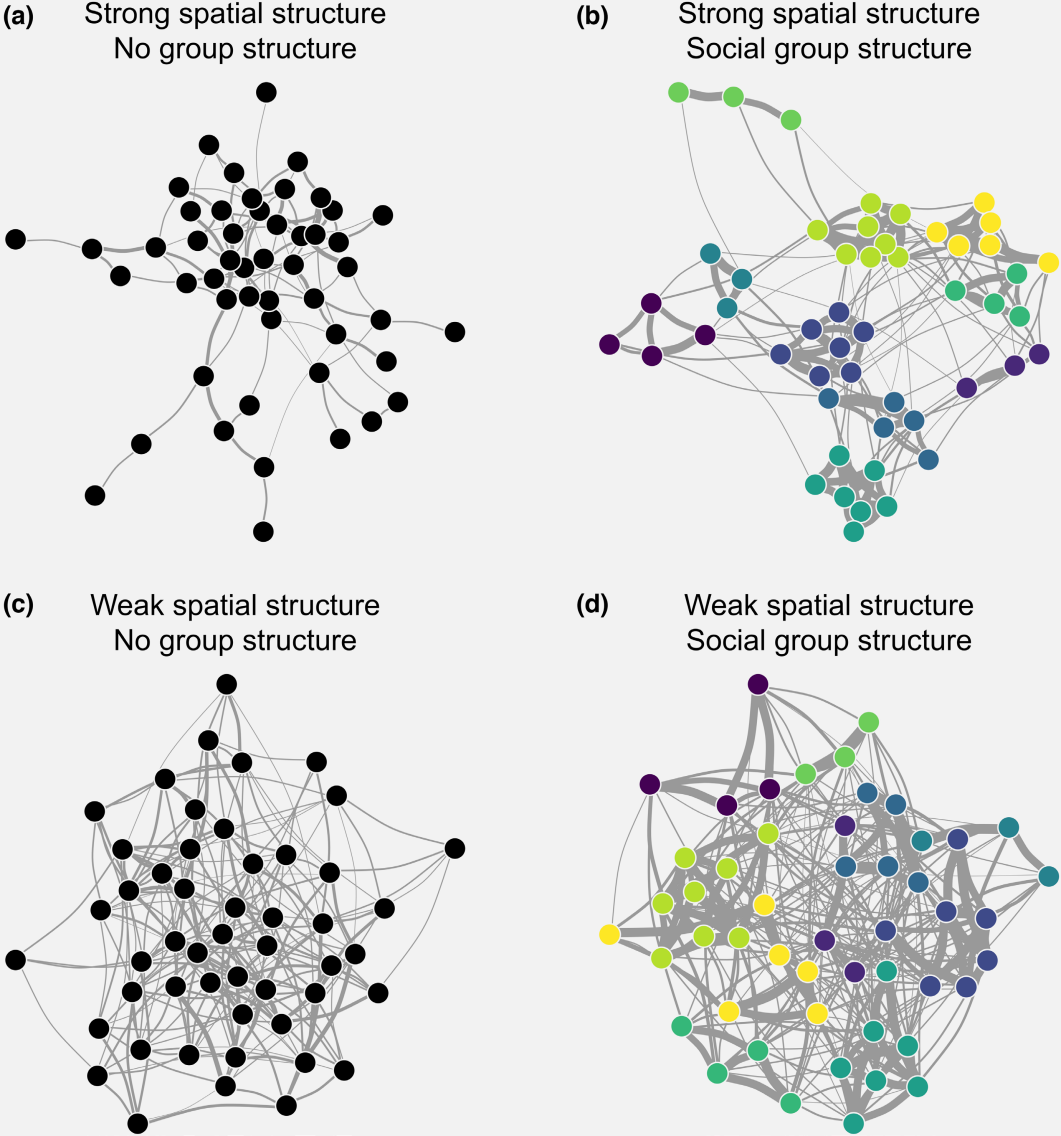
Examples of the diverse underlying social network structures it is possible to simulate with genNetDem. Here, we explore the impact of spatial and social structure on otherwise similar sets of rules for the generation of social relationships. Code to replicate this figure and further explore network possibilities is provided in the Appendix S3.

The **network_generation_covariates()** function generates an underlying network structure based on social group membership (as defined when generating the population), spatial locations and individual traits. Figure 2 shows examples of networks generated. Current functionality is focused on how these traits impact the probability of forming social connections within and between groups separately, thus employing an adapted stochastic block model (Lee & Wilkinson, 2019). Block membership is defined based on the assignment of individuals to prespecified social groups. It is additionally possible for between block edge probabilities to be further modified by the spatial distance between groups (Figure S13; the spatial structure ‐ implemented by multiplying baseline values for between‐block edge probabilities and weights by ${\frac{1}{d\_\mathrm{eff}}}^{\text{distance}}$, where d_eff is a user specified effect and distance is the distance between groups). Therefore, genNetDem is not directly designed to incorporate some known social processes such as triadic closure or assortativity, for example females being more closely connected to other females, although it could be possible to use group membership and no spatial structure to approximate these effects (and it is also important to note that assortativity or triadic closure can also arise [indirectly] as an emergent property of the selected generative model). It is also currently not possible for interaction effects to be coded directly (e.g., if size effects on connectivity were different for males and females). Edge probabilities and edge weights are modeled independently to allow variables to explain variation in one or both of them. Edge weights are parameterized by fitting a beta distribution to a provided mean and variance, generating edge weights between 0 and 1 in the underlying network. There is also a simpler **network_generation_basic()** function that uses the same generative model without covariates.

When modeling longitudinal network data, the individual social network positions could vary from relatively stable to highly dynamic (Pinter‐Wollman et al., 2013). The **network_rewire_covariates()** function adds newly recruited and removes dead individuals from the network. It also provides functionality to select probabilities that (a) an individual changes some social relationships, and (b) each social relationship for selected individuals changes. Rewiring of edges uses the same generative model as the initial generation of networks. Thus, it is possible to parameterize network_rewire_covariates() such that new social connections follow the same rules as others in the network or to simulate different network structures (e.g., reducing the importance of social group membership or spatial structure). This allows flexibility in how dynamic simulated networks are.

There are two functions that generate grouping events based on underlying network structure: **interaction_generation_simul()** and **interaction_generation_seq()**. The difference between them is that the former divides all individuals in the population into grouping events (or isolates) at each time point, while the latter independently samples one grouping event of a defined size from the population at a time. The former is more widely useful. It uses data on individual IDs, their underlying social network and a mean grouping event size to divide the populations into events, with grouping event membership being stored in a group‐by‐individual matrix (GBI; see Farine, 2013). The n_ts argument defines the number of times this process is repeated (i.e. the number of “***behavioral timesteps***”). Assigning individuals into grouping events based on the underlying network can create computational challenges if unconstrained. We use a similar approach as previously described (Evans et al., 2020), with individuals being added to events sequentially and the probability of joining being proportional to the strength of its social relationships with existing members calculated using ${\left(\text{product of edge weights}+\frac{\mathrm{sum}\;\text{of edge weights}}{\mathrm{pm}}+\text{float}\right)}^{\mathrm{pow}}$ (where pm adjusts the relative importance of the edge weight sum versus the product, float prevents all joining probabilities being zero and pow adjusts the final joining probabilities; see Appendix S2). Including a nonzero float argument means it is never impossible to add an individual to an existing grouping event even in the absence of any social connections. While it may be tempting to reduce the float to zero, this can result in it being impossible (or computationally challenging) to successfully sample all individuals into grouping events. However, care should be taken with particular combinations of grouping event size distributions and underlying network structures that these relaxations do not dominate grouping event generation. This can be checked with the **network_checker_simul()** function. The network_checker_simul() makes it possible to compare network measures calculated from the network generated from grouping event data with those calculated from the underlying network, and uses the netlm function from sna (Butts, 2014) to conduct a matrix regression between the two networks to test the association between edge weights in each (see Appendix S2 for more detail).

### 4.4 Observation features

The observation features sample the simulated grouping events and generate data for subsequent analyses. Data are generated in a variety of formats including GBI matrices for social network analysis and classic capture–recapture formats. There are two **cap_and_obs()** functions that generate an observed network dataset based on the sampling strategy and design. Of the two **cap_and_obs2()** has greater flexibility (see Appendix S2). Inputs include: (a) data on true grouping events (the GBI and a vector indicating which behavioral timestep each grouping event occurred in); (b) vectors indicating behavioral timesteps to be sampled, indicated separately for captures and observations; (c) the success of sampling including both the proportion of grouping events detected and the proportion of individuals in each sampled event detected; and (d) a vector indicating which (if any) individuals had been captured previously. The function then samples grouping events from each behavioral timestep indicated for captures and observations using a predefined probability (pcg and pmg, respectively), and then individuals within these grouping events with a second predefined probability (pci and pmi, respectively). Captures take precedence over observations in behavioral timesteps where both are indicated. Individuals can only be observed whether they have previously been captured (although it is possible to provide additional information on previous captures using the argument pre_cap). The function returns GBIs for captured and observed groups and other related information. The **cap_dat_gen()** function transforms these network datasets into capture histories for both behavioral timesteps and demographic timesteps and the **obs_net_checker()** function provides comparisons between sampled networks and both the network derived from grouping event data and the underlying population network.

## 5 CASE STUDIES

We use two complementary case studies to illustrate the use of genNetDem. In the first, we test how our ability to estimate the relationship between network position and survival depends on sampling effort; whether local or global centrality affects survival; and network dynamics. We compare the performance of cross‐sectional versus longitudinal imputation of the network position of nondetected individuals and explore the importance of network covariance in survival probabilities. In the second, we demonstrate how a researcher could use genNetDem to compare sampling designs. We test how the power to estimate relationship between network position and survival depends on how sampling effort is distributed through time. Our simulation asks the question as to whether it is better to concentrate resources into intensively monitoring more groups in fewer sampling windows or fewer groups in more sampling windows. We examine whether any differences are impacted by the proportion of individuals detected in each sampled group and the structure of the underlying social network.

### 5.1 Methods common to both case studies

In both case studies, we use genNetDem to simulate survival and social interactions and then sample from them to generate capture histories. Illustrations of the workflows used a provided in Figures S1 and S2. We fit HMMs to estimate survival and capture probabilities using nimble (de Valpine et al., 2017, 2022).

#### 5.1.1 Data recorded from simulation runs

We recorded (a) the capture–recapture dataset for each demographic timestep; (b) the sampled social network generated from all observed interactions within each demographic timestep; (c) individual survival probabilities for each demographic timestep; and (d) information on true population size and the number of individuals recorded at each demographic timestep. We estimated the network measure of interest from the sampled social network and scaled it (mean‐centered and scaled to have unit variance) within each demographic timestep to use as an explanatory variable.

#### 5.1.2 Modeling approach

We fitted CJS models estimating both capture and survival probabilities (Lebreton et al., 1992) and used Bayesian inference for parameter estimation. We included explanatory variables of sex and social network measure (either strength or betweenness). In each model, we used weakly informative priors for all parameters (Gaussian distribution with *μ* = 10 and *σ* = 10 for survival‐related variables, uniform distribution between 0 and 1 for capture probability). We used a single Markov chain of 3000 iterations with a burn‐in of 500 and a thinning interval of 5. We confirmed that this number of iterations was typically sufficient for model convergence and an adequate effective sample size in a subset of simulations.

#### 5.1.3 Analysis of simulation results

From each simulation run, we calculated the posterior median and standard deviation, the proportion of the posterior >0, and the 89% HDI. We also calculated a binary variable indicating whether or not 0 was contained within the 89% HDI. We could then compare model performance visually and by calculating statistical clarity for positive social effects on survival as the proportion of simulation runs where 0 fell outside the 89% HDI.

### 5.2 Case study 1: Performance of basic imputation to estimate social effects on survival

#### 5.2.1 Specific methods

##### 5.2.1.1 Overview of data generation

We simulated a population of 200 individuals with no underlying social group structure. Individual variation was restricted to a single two‐level categorical variable—sex (allocated stochastically; each individual had a 50% chance of being either male or female). The underlying social network had moderate spatial structure.

We simulated the behavior and survival of individuals over 10 demographic timesteps (over which survival was simulated), each containing five behavioral timesteps (at which individuals were organized into grouping events). Grouping events had a mean size of two individuals (many events were dyadic and individuals were frequently alone) to capture a situation where a species rarely occurs in large aggregations. Survival probability depended on sex (moderate effect of 0.5 on a logit scale) and position in the social network calculated from grouping events (see below) with a baseline survival probability of 0.8 in females. We assumed no recruitment into the population (i.e., the population declined over the simulation).

We assumed that all individuals in the population were marked or individually identifiable prior to the start of the study. Captures and/or observations (which were functionally equivalent as all individuals were marked) took place in all behavioral timesteps (50 in total). Each grouping event had either a 25%, 50%, or 75% percent chance of being detected (parameter varied between simulation runs) with the detection probability of an individual in a detected group fixed at 0.9.

##### 5.2.1.2 Simulation structure

In total, we generated 3240 simulated datasets, varying five parameters that influenced network dynamics (one parameter), network effects on survival (three parameters), and sampling (one parameter).

- *Network dynamics*: we varied the probability that an individual's existing connections in the underlying social network were rewired after each demographic timestep with values set at 0 (no rewiring), 0.1, and 0.5. If it did rewire its connections, then the per‐edge probability that an individual changed its connections was 0.5. Edges were rewired using the same generative model used to create the initial network.
- *Network effects on survival*: (a) we varied the network measure that influenced survival to be either strength (local measure; sum of weighted connections) or betweenness (global measure; number of shortest paths passing through an individual); (b) we varied the effect size to be 0 (no effect), 0.4 (moderate effect) or 0.8 (strong effect); (c) we altered covariance of individual survival within the network to be negative (individuals strongly connected with each other have more dissimilar survival probabilities), neutral or positive (strongly connected individuals have more similar survival probabilities).
- *Sampling*: we varied the probability of sampling (either capturing or observing) a group at each behavioral timestep to be 0.25, 0.5, or 0.75.

An illustration of the workflow used is in Figure S1. For each combination of parameters (162), we ran 20 replicates.

##### 5.2.1.3 Data recorded

In addition to the four types of data described in the combined methods, we also recorded the full social network generated from all interactions within each demographic timestep (including those not observed). We estimated the network measure of interest from these full networks and scaled them within each demographic timestep as for measures from partial networks.

##### 5.2.1.4 Model‐fitting

From each simulation run, we fitted four model versions (see combined methods for details on model‐fitting). The four versions differed in: (a) using the measure from the sampled network and a longitudinal approach for imputing nonobserved individuals; (b) using the measure from the sampled network and cross‐sectional imputation; (c) using the measure from the full (unobserved) network and longitudinal imputation; and (d) using the measure from the full network and cross‐sectional imputation. For cross‐sectional imputation, missing values were estimated using the mean and variance of the (scaled) focal network measure for all individuals from a given demographic timestep. For longitudinal imputation, missing values were estimated using the mean and variance of the focal network measure for each individual across all timesteps in which it was captured or observed where possible and the overall mean and variance when not (i.e., when an individual was only captured once).

##### 5.2.1.5 Analysis of simulation results

Prior to the general analysis outlined above, we assessed whether the model had converged using the posterior median and standard deviation of its estimate for the social effect on survival. We used *k* means clustering to identify groups of simulation runs where the model was unlikely to have converged. We used *k* = 6 clusters and retained three of six of these clusters based on the elbow method and visual inspection of the output (Figure S3). This method identified ~2.5% of models had likely not converged.

To compare the success of models that used network measures calculated from the partial versus full network, we calculated the earth mover's distance (EMD) of the posterior distributions (Touzalin et al., 2022) for the parameter of interest from relevant pairs of models. (i.e., we calculated the EMD for model versions using the full and partial network together with longitudinal imputation and also the EMD for the model versions using the full and partial network together with cross‐sectional imputation). Earth mover's distances provide a measure of overlap of the posterior distributions.

#### 5.2.2 Results and discussion

**FIGURE 3:**
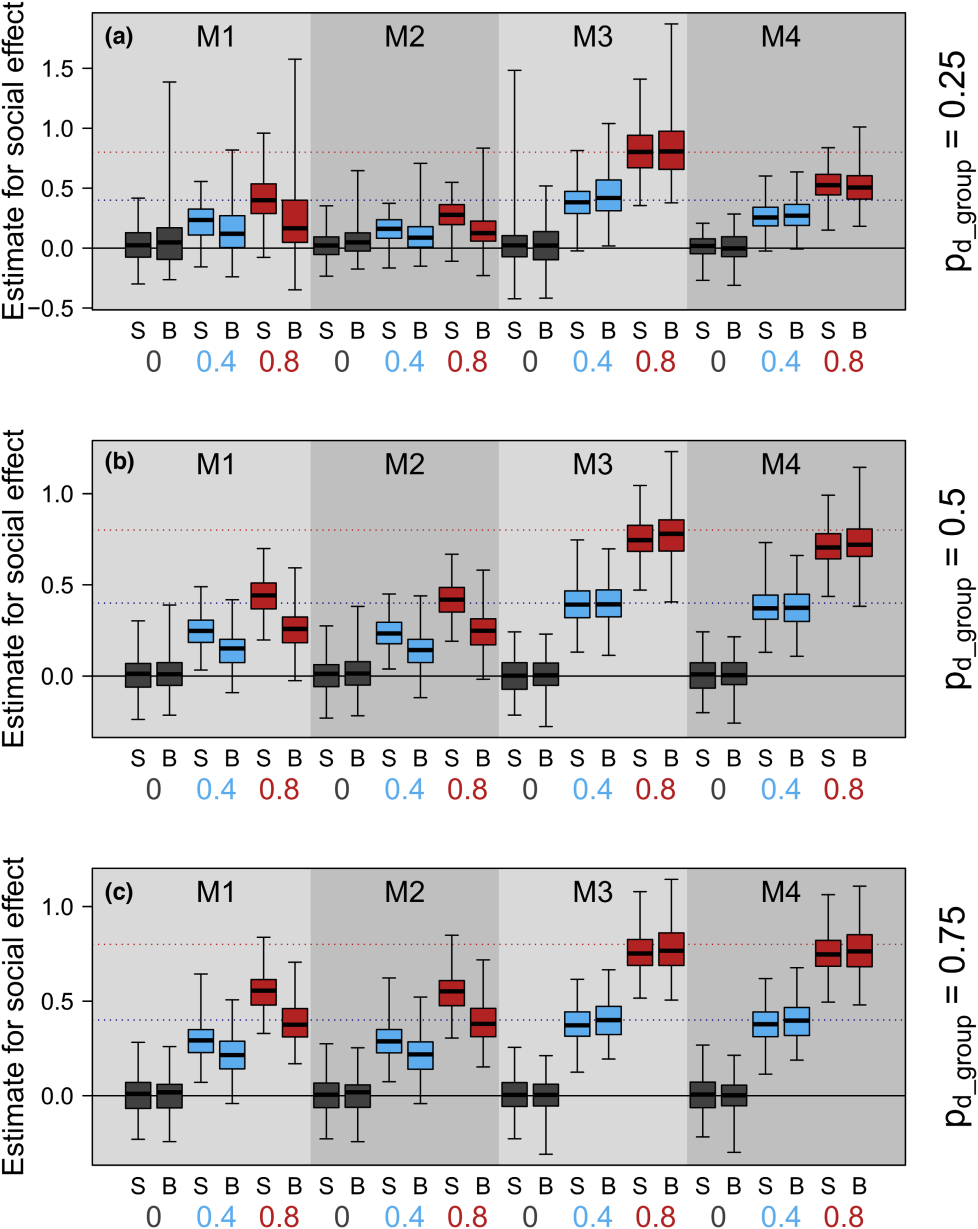
Distribution of posterior medians for the social effect of survival for different combinations of model (shaded polygons; M1: partial network—cross‐sectional imputation; M2: partial network—longitudinal imputation; M3: full network—cross‐sectional imputation; M4: full network—longitudinal imputation), network measure (S = Strength; B = Betweenness) and true effect size (box color) when (a) 25% of groups are sampled, (b) 50% of groups are sampled and (c) 75% of groups are sampled. The solid central line represents the median, boxes the interquartile range and whiskers the full range of values. The blue‐dotted line indicates the accurate parameter estimate when the true effect size is 0.4 and the red‐dotted line when the true effect size is 0.8.

Overall, we show it is possible to estimate social effects survival from partial networks, albeit with substantial limitations in power (Figure 3, Table 2; Table S1). Estimates of social effects on survival were downward biased meaning that statistical power was limited and only stronger social effects on survival are likely to be detected. Sampling effort was particularly important and interacted with how imputation was conducted in determining how well models converged and biases in parameter estimates when they did. Estimates of other parameters were unaffected.

**TABLE 2:** Proportion of simulation runs where 0 falls outside the 89% HDI for different parameter combinations.

Network measure	True effect	Model	Group capture probability	Detection rate
Strength	0.4	M1	0.50	0.75
Strength	0.4	M2	0.50	0.72
Strength	0.4	M3	0.50	0.98
Strength	0.4	M4	0.50	0.98
Betweenness	0.4	M1	0.50	0.26
Betweenness	0.4	M2	0.50	0.25
Betweenness	0.4	M3	0.50	0.94
Betweenness	0.4	M4	0.50	0.94
Strength	0.8	M1	0.50	0.99
Strength	0.8	M2	0.50	0.99
Strength	0.8	M3	0.50	1.00
Strength	0.8	M4	0.50	1.00
Betweenness	0.8	M1	0.50	0.70
Betweenness	0.8	M2	0.50	0.68
Betweenness	0.8	M3	0.50	1.00
Betweenness	0.8	M4	0.50	1.00

*Note*: M1: partial network—cross‐sectional imputation; M2: partial network—longitudinal imputation; M3: full network—cross‐sectional imputation; M4: full network—longitudinal imputation.

Previous research has demonstrated that network measures from sampled, partial networks are correlated with those in the full, unobserved network but that these correlations vary depending on the proportion sampled and network measure calculated (Silk et al., 2015; Smith & Moody, 2013). Furthermore, the regression slope is rarely 1:1 indicating values for measures estimated are not perfectly accurate (Silk et al., 2015). This likely explains many of our results showing the difficulty of detecting social effects on survival in the absence of network imputation or the use of measures from independently (and better) sampled social networks.

##### 5.2.2.1 Network variable and covariance structure

When we compared models that used network measures from the full and partial networks, we found downward‐biased parameter estimates and reduced statistical clarity of results when partial network measures were used (Table 2, Figure 3). These patterns were more striking when survival was related to a global measure of centrality (betweenness) than a local measure of centrality (strength). We found that including positive or negative covariance in survival probabilities related to social network structure had little effect on estimation or power in the contexts simulated (Figure S5, Tables S5 and S6).

These results fit well within the literature on how missing individuals impact the conclusions of social network analysis, with previous studies showing that global estimates of social centrality (such as betweenness) from partial networks are less well‐correlated than measures of local centrality (such as strength) with equivalent measures from the full network (Silk et al., 2015). While for strength in particular, downward‐biased parameter estimates in combination with maintained statistical power could also be related to measures of strength being lower in the smaller, sampled network (Silk et al., 2015); this should be controlled for by scaling network measures before using them in the model. The lack of a clear effect of network covariance is somewhat surprising. These results are promising in suggesting that this may present a more limited issue in this context than often considered (e.g., Croft et al., 2011; Farine & Carter, 2020; Silk et al., 2017). However, the importance of covariance likely depends substantially on network structure and density, so it would be unwise to generalize these patterns without further work focused specifically on this question.

##### 5.2.2.2 Sampling effort, imputation approach, and network dynamics

Lower sampling effort was typically associated with both (a) reduced likelihood of model convergence (Table 3; Table S2), and (b) downward‐biased parameter estimates (Figure 3). However, the nature of these relationships depended on the imputation approach selected (Table 2, Figure 3), with the performance of different imputation approaches largely independent of network dynamics (Tables S3 and S4, Figure S4).

**TABLE 3:** Convergence rates of models using different imputation approaches for various parameter combinations.

Network measure	Model	Group capture probability	Convergence rate
Strength	M1	0.25	0.98
Strength	M1	0.50	0.99
Strength	M1	0.75	1.00
Strength	M2	0.25	1.00
Strength	M2	0.50	1.00
Strength	M2	0.75	1.00
Strength	M3	0.25	1.00
Strength	M3	0.50	1.00
Strength	M3	0.75	1.00
Strength	M4	0.25	1.00
Strength	M4	0.50	1.00
Strength	M4	0.75	1.00
Betweenness	M1	0.25	0.65
Betweenness	M1	0.50	0.94
Betweenness	M1	0.75	0.98
Betweenness	M2	0.25	0.96
Betweenness	M2	0.50	1.00
Betweenness	M2	0.75	1.00
Betweenness	M3	0.25	0.96
Betweenness	M3	0.50	0.97
Betweenness	M3	0.75	0.99
Betweenness	M4	0.25	1.00
Betweenness	M4	0.50	1.00
Betweenness	M4	0.75	1.00

*Note*: M1: partial network—cross‐sectional imputation; M2: partial network—longitudinal imputation; M3: full network—cross‐sectional imputation; M4: full network—longitudinal imputation.

Models were much less likely to converge when sampling effort was low (25% group capture probability), betweenness centrality from partial networks was used as an explanatory variable and cross‐sectional imputation was used to infer missing values (Table 3; Table S2). Even when 50% of groups were sampled in these situations, there was still a reduction in convergence rate. Note that this was apparent regardless of whether betweenness centrality had a positive or no effect on survival probability. Any other changes in the likelihood of model convergence were of much smaller magnitude, but generally occurred when sampling effort was low (and measures from partial networks were used).

With cross‐sectional imputation and use of measures from the full network, estimation of social effects on survival were largely independent of sampling effort in the contexts examined. With longitudinal imputation, there was some reduction in estimates of the social effect on survival with low levels of sampling (25% groups sampled). However, both cross‐sectional and longitudinal imputation demonstrated similar relationships between sampling effort and statistical power (Table 2; Table S1), indicating that posterior distributions had higher variance when cross‐sectional imputation was used.

**FIGURE 4:**
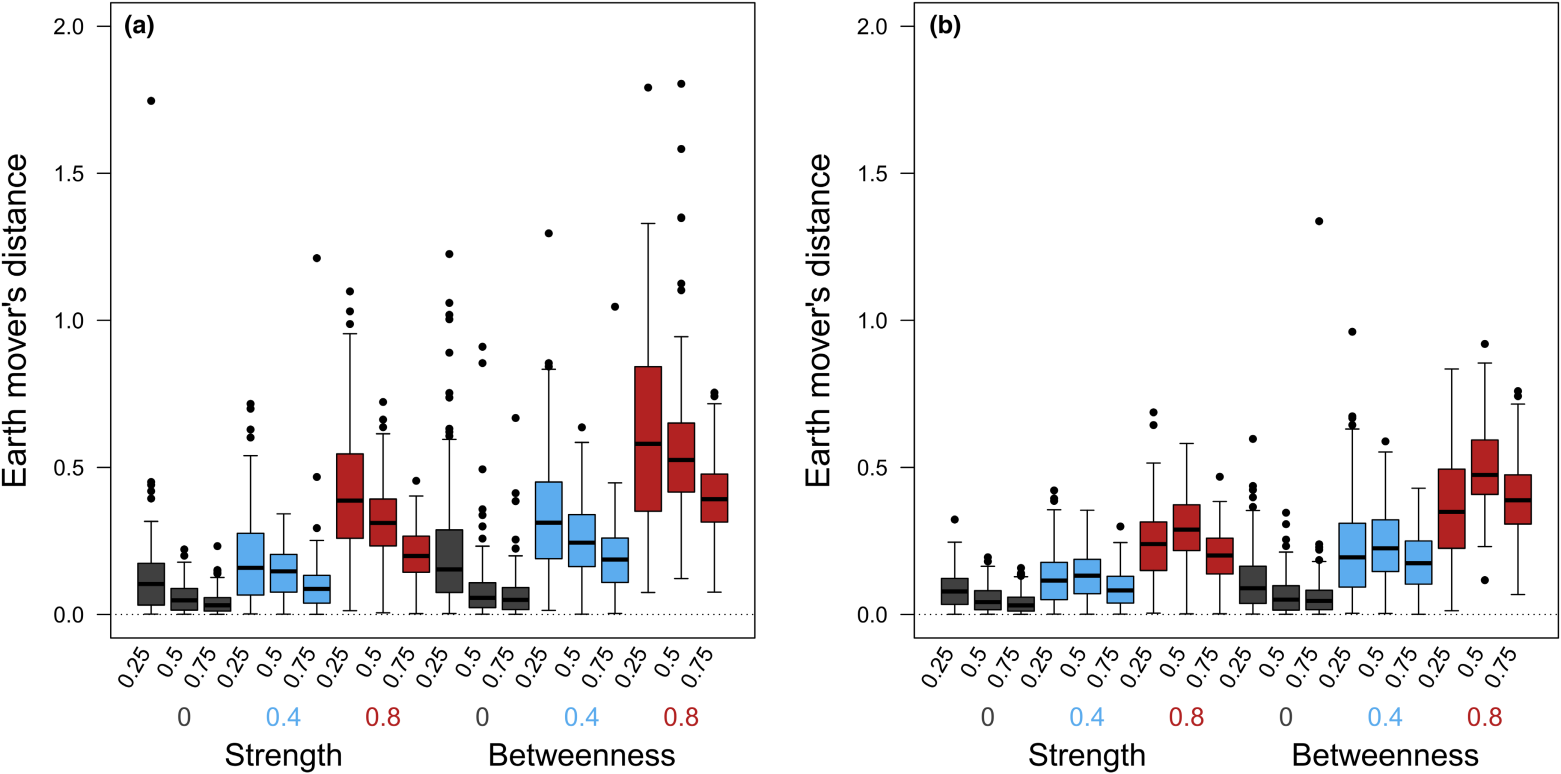
Earth mover's distances demonstrating the similarity of the posterior distributions for model estimates of the social effect on survival between the model using full and partial network measures for (a) cross‐sectional and (b) longitudinal imputation. The solid central line represents the median, boxes the interquartile range and whiskers extend to 1.5× the interquartile range. We show the distributions for different combinations of group capture probability (0.25, 0.5 or 0.75), true simulated effect size (gray for 0, blue for 0.4 and red for 0.8) and the network measure influencing survival probability (strength vs. betweenness). To aid visualization we have excluded 19 outlying points with EMD > 2 for panel a).

When measures from the partial network were used instead, there was a much more substantial reduction in both parameter estimates and statistical power apparent even for higher sampling efforts (Figure 3, Table 2). Reductions in parameter estimates were more substantial and remained linear when longitudinal imputation was used, instead flattening out for cross‐sectional imputation so that the difference between 25% and 50% of groups being sampled was less than the difference between 50% and 75% (Figure 3). However, similarly to the pattern for full network measures, this was not reflected in changes to statistical power which were broadly equivalent for both, indicating a less precise posterior distribution for cross‐sectional imputation. These differences between cross‐sectional and longitudinal imputation changed how EMDs calculated for the differences between posteriors from the full network and partial network model fits depended on sampling effort (Figure 4). For cross‐sectional imputation, EMDs were highest for low sampling effort (*p* = .25) while for longitudinal imputation, they peaked at intermediate sampling effort (*p* = .5). However, in general, EMDs were higher for cross‐sectional than longitudinal imputation.

Our results show that when social networks are constructed based on the same co‐capture data used to estimate survival, even relatively small drops in sampling effort can lead to downward biases in parameter estimates and statistical power. While this pattern was especially strong when global measures of centrality such as betweenness explain variation in survival probability as expected from previous literature (Silk et al., 2015; Smith & Moody, 2013), it was also apparent when strength was associated with survival instead. However, in this latter case, underestimated social effects on survival only caused substantial reductions in statistical power with very low sampling effort. Consequently, our results fit broadly within the existing literature where low sampling effort has a greater impact on global measures of centrality but suggest that missing a high proportion of interaction events leads to wider problems with subsequent statistical analyses. This was particularly apparent when cross‐sectional imputation was used to estimate missing values for betweenness centrality, in which case there was a substantial drop‐off in how likely models were to converge. Combined with cross‐sectional imputation generating less precise posteriors, this suggests that longitudinal imputation is the more stable option of the two, although it does lead to greater downward bias in estimates of social effects on survival. However, neither imputation approach performed well, highlighting the value of extending network imputation approaches (Krause et al., 2018, 2020; Young et al., 2020) within capture–recapture models. A good example is provided by Clements et al. (2022), who estimate not only the network itself but also the underlying behaviors that generate the network structure within a CJS model. While this was done in the context of a simulation study, and so involved fitting the data‐generating model, it does show the potential of network imputation to improve the accuracy of estimates of social effects on survival.

##### 5.2.2.3 Estimates of other parameters

Estimates for other parameter values were unaffected by social effects on survival, use of measures from full or partial networks or imputation strategy (Figures S6–S8).

### 5.3 Case study 2: Effective sampling strategies to estimate social effects on survival

#### 5.3.1 Specific methods

##### 5.3.1.1 Overview of data generation

We simulated a population of 200 individuals with either (a) no underlying social group structure or (b) divided into 20 social groups. Individual variation in the population was restricted to a single two‐level categorical variable—sex. Underlying network structure depended on parameter choice (see below).

We simulated the behavior and survival of individuals over 10 demographic timesteps, each of which contained 20 behavioral timesteps. As previously, grouping events had a mean size of two individuals. The survival probability of each individual depended on its sex (fixed effect of 0.5 on a logit scale) and position in the social network calculated from grouping events (see below), with a baseline survival probability of 0.8 in females. In this case study, there was recruitment into the population over time (i.e., the population stayed roughly constant over each simulation). There was a 10% chance that a surviving individual rewired its underlying social connections after each demographic timestep, and if it did each connection had a 50% chance of changing. Edges were rewired using the same generative model used to create the initial network.

The population was initially unmarked. Captures only occurred in the first behavioral timestep of each demographic timestep with 90% of groups sampled and a 0.9 probability of individuals in a sampled group being detected. Sampling design and effort for subsequent observations depended on parameter choice (see below).

##### 5.3.1.2 Simulation structure

In total, we generated 2880 simulated datasets, varying five parameters that influenced network structure (one parameter), network effects on survival (two parameters), and sampling effort/design (two parameters).

- *Network structure*: we varied underlying network structure so that either (a) there was no group structure and moderate spatial structure driving the probability and weight of edges or (b) the population was divided into 20 groups with the probability of a within‐group connection of 0.5 and within‐group connection weights having a mean of 0.5 (vs. a baseline of 0.2 and 0.25, respectively, for between‐group connections prior to adjusting for distance effects).
- *Network effects on survival*: (a) we varied the network measure that influenced survival to be either strength or betweenness and (b) we varied the effect size to be 0 (no effect), 0.4 (moderate effect), or 0.8 (strong effect).
- *Sampling*: (a) we varied sampling design so that the probability of observing a grouping event within a sampled behavioral timestep covaried with the number of behavioral timesteps sampled in each demographic timestep resulting in (approximately) equivalent sampling effort being divided over the full demographic timestep. The probability of observing an event was either 0.1, 0.2, 0.4, or 1, with the number of behavioral timesteps observed being 19, 10, 5, or 2; (b) we varied the probability of an individual being observed in a sampled group to be either 0.5, 0.75, or 1.

An illustration of the workflow used is in Figure S2. For each combination of parameters (144) we ran 20 replicates.

##### 5.3.1.3 Model‐fitting

Unlike Case Study 1, each CJS model was conditioned on first capture (as individuals were not assumed to have been captured previously).

#### 5.3.2 Results and discussion

**FIGURE 5:**
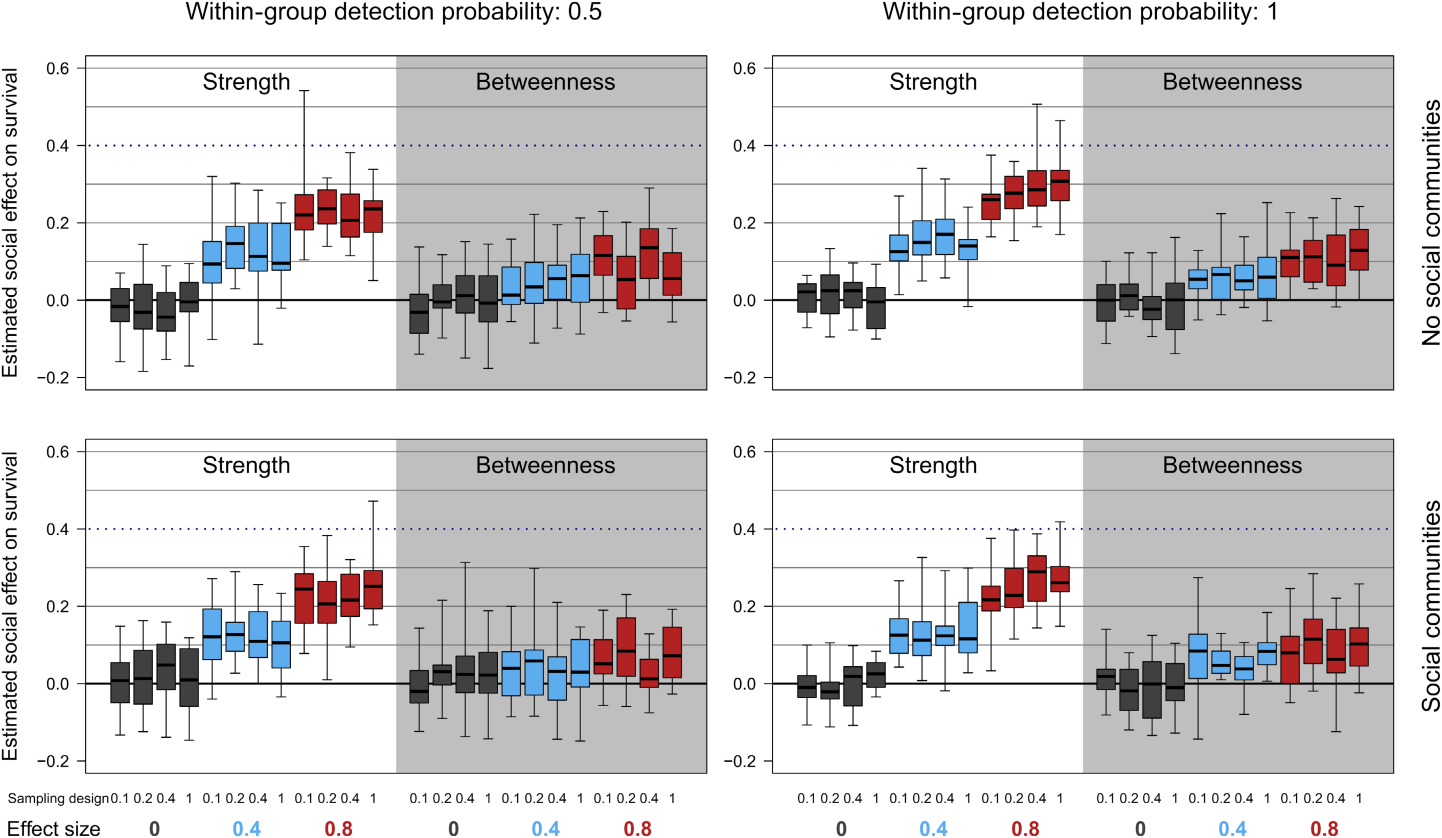
Impacts of sampling design (within‐plot: sets of boxes of the same color), within‐group detection probability (columns) and social structure (rows) on Cormack‐Jolly‐Seber estimates of social effects on survival probability for a range of simulated effect sizes (colors of boxes). Boxplots show the distribution of posterior medians from multiple simulation runs with the solid line the median, boxes the interquartile range and whiskers the full range of values. We illustrate contexts in which a local measure of centrality (strength) and global measure of centrality (betweenness) are used as explanatory variables. The blue‐dotted line indicates the accurate parameter estimate when the true effect size is 0.4 (the equivalent line for 0.8 is not illustrated).

Overall, survival models performed adequately in detecting social effects on survival (Table 4, Figure 5; Tables S7–S9, Figure S9). When we simulated positive effects of network centrality on survival probabilities model estimates reflected this, although were substantial underestimates, especially with only moderate social effects on survival. These results support those from Case Study 1 indicating that it is possible to estimate social effects on survival, but that statistical power is limited with the presence of nondetected individuals and/or when many grouping events are unobserved. More encouragingly, we show that for two very different social network structures there is little evidence for strong bias or elevated false‐positive rates when there is no social effect on survival.

**TABLE 4:** Proportion of simulation runs where 0 falls outside the 89% HDI for different parameter combinations with the probability of within‐group detection fixed at 1.

Network measure	True effect	Social structure	Sampling design	Detection rate
Strength	0.4	Communities	0.1	0.45
Strength	0.4	No communities	0.1	0.50
Strength	0.4	Communities	0.2	0.40
Strength	0.4	No communities	0.2	0.70
Strength	0.4	Communities	0.4	0.55
Strength	0.4	No communities	0.4	0.70
Strength	0.4	Communities	1.0	0.50
Strength	0.4	No communities	1.0	0.60
Betweenness	0.4	Communities	0.1	0.20
Betweenness	0.4	No communities	0.1	0.10
Betweenness	0.4	Communities	0.2	0.00
Betweenness	0.4	No communities	0.2	0.10
Betweenness	0.4	Communities	0.4	0.00
Betweenness	0.4	No communities	0.4	0.15
Betweenness	0.4	Communities	1.0	0.10
Betweenness	0.4	No communities	1.0	0.15
Strength	0.8	Communities	0.1	0.95
Strength	0.8	No communities	0.1	1.00
Strength	0.8	Communities	0.2	1.00
Strength	0.8	No communities	0.2	1.00
Strength	0.8	Communities	0.4	0.95
Strength	0.8	No communities	0.4	1.00
Strength	0.8	Communities	1.0	1.00
Strength	0.8	No communities	1.0	1.00
Betweenness	0.8	Communities	0.1	0.15
Betweenness	0.8	No communities	0.1	0.35
Betweenness	0.8	Communities	0.2	0.45
Betweenness	0.8	No communities	0.2	0.45
Betweenness	0.8	Communities	0.4	0.25
Betweenness	0.8	No communities	0.4	0.40
Betweenness	0.8	Communities	1.0	0.40
Betweenness	0.8	No communities	1.0	0.50

##### 5.3.2.1 Network variable

Our statistical models were better able to detect the effect of strength (local centrality measure) than betweenness (global centrality measure) on survival probabilities. While the effect size was underestimated for both measures, this bias was much greater for betweenness centrality (Figure 5), and results were more frequently statistically unclear (Table 4). The results here support those from Case Study 1 and the existing literature (Silk et al., 2015; Smith & Moody, 2013) in highlighting that global measures of network position are more susceptible to sampling effects than local measures.

##### 5.3.2.2 Sampling design

There was no clear effect of how grouping events were sampled within each demographic timestep on estimates of social effects on survival (Figure 5). Unsurprisingly, probability of observing individuals within groups did have some effect, with less downward‐biased parameter estimates and more statistical power when sampling within groups was more complete (Figure S9, Tables S7 and S8), as would be expected.

Lower observation success within sampled grouping events leading to reduced model performance is unsurprising as it leads to missing edges in the sampled network, reducing its correlation with the true (unobserved) network. This finding supports related work focused on calculating network measures (e.g., Franks et al., 2010). Franks et al. (2010) also tentatively recommended that more censuses (behavioral timesteps sampled in our case) were preferable than ensuring a high proportion of grouping events sampled in each census for calculating weighted measures of centrality. However, we found no clear evidence that this extended to our survival analysis, where there were only small differences in model performance and no clear overall trend. It should be noted, however, that the simulation architecture differed between the two papers.

##### 5.3.2.3 Social structure

Social structure had a small effect on the ability to detect social effects on survival, with some differences in statistical power between the two structures investigated. While there were minimal differences in posterior medians (Figure 5), results tended to be statistically clearer when there was no underlying group structure than when the population was divided into 20 groups (Table 4). Previous studies of sampling in social networks have rarely considered the types of modular social structures common for group‐living animal populations (Silk, 2018). The slight negative impact of this group‐structure on our ability to detect social effects on survival perhaps suggests that the correlation between network measures calculated in the sampled and full networks is weaker in these types of networks.

##### 5.3.2.4 Estimates of other parameters

Estimates of other parameters were largely unaffected by social effects or sampling design. Strong social effects on survival were associated with slightly lower estimates of mean survival probability, but these differences were caused by differences in simulated survival probabilities rather than model performance (Figures S10–S12). While limited in scope, these results provide evidence that including social effects on survival in demographic models is unlikely to impact other parameter estimates substantially (see also Clements et al., 2022).

## 6 FUTURE STEPS

With the two case studies presented, we only scratch the surface of the potential of genNetDem as a methodological tool for animal social network analyses. Below, we highlight some logical next steps for methodological studies on this topic, focusing on the integration of social networks and demography.

First, while we demonstrated the capacity for genNetDem to generate diverse social structures (Figure 2), this was only a partial focus of our results. Animal social systems vary widely, and while optimal sampling strategies are likely to vary with social structure (Clements et al., 2022; Silk, 2018; Sunga et al., 2021), this has remained understudied. Similarly, while we varied network dynamics in our simulations, individual variation in edge probabilities was limited. Incorporating greater trait‐based or individual variation into network position would likely influence conclusions drawn about imputation approaches, for example. The modular design of genNetDem allows it to be integrated with other tools to simulate social network structure (e.g., Ross et al., 2022), which will help tackle these types of challenges more comprehensively in future.

Second, it is clear that simple approaches to imputing missing network measures are only partially successful; while they successfully generate qualitatively correct results, parameter estimates for social effects on survival are underestimated. Although developing more sophisticated approaches to impute values for network measures may help, exploiting recent developments in network imputation (Krause et al., 2018, 2020; Young et al., 2020) is likely to have the greatest success. The adaptation of these novel approaches for behavioral ecology, and specifically within this capture–recapture modeling framework is a key challenge. Gimenez et al. (2019) applied basic network imputation to study the social structure of Commerson's dolphin *Cephalorhynchus commersonii*. Similarly, Clements et al. (2022) included estimation of network structure within a CJS model to improve estimation of social effects on survival. However, the latter approach used a rather basic generative model for the latent network structure that could be improved on or adjusted for researchers working in different contexts. Consequently, extending these approaches to incorporate more sophisticated social network models as well as to open populations is a key priority.

Third, to keep our case studies accessible, we examined social effects only in CJS models to estimate survival probability. Clements et al., (2022) highlighted the potential value of incorporating social networks within integrated population models (IPMs), where different data sources could also be used to inform network structure itself. However, especially with improvements to imputation of latent network structures, there is also great potential to incorporate network effects within multistate models more generally. Given the central role of social behavior in mediating interactions between infectious disease dynamics and demographic processes (Silk et al., 2019; Silk & Fefferman, 2021), extending multistate models to incorporate social network structure in this way could provide important new insights into wildlife disease ecology, to provide just one example. genNetDem can provide an ideal sandbox to refine these models for application to wild systems.

Finally, we focus here on dyadic social networks; however, many of the social interactions studied are nondyadic and may include higher‐order interactions (Battiston et al., 2021; Greening Jr et al., 2015). While there has been limited focus on higher‐order interactions in animal societies (Musciotto et al., 2022), theory suggests they will impact infectious disease transmission and social contagions (Battiston et al., 2021; Iacopini et al., 2022; Noonan & Lambiotte, 2021) among other ecological and evolutionary processes. Therefore, expanding some of the developments here beyond dyadic networks to consider higher‐order effects on survival and imputation of hyperedges (social connections between more than two individuals) will likely represent valuable developments. Because it generates GBIs that incorporate interactions/associations between more than two individuals, genNetDem is an ideal starting point for methodological research testing higher‐order methods in animal societies.

## 7 CONCLUSIONS

We introduce the R package genNetDem as a flexible tool for simulating combined social and demographic datasets. While we focus on the integration of social network and demographic models, the modular design of the package allows it to be an equally powerful tool for generating social network or capture–recapture datasets in their own right. It therefore provides a general tool for researchers interested in testing key methodological considerations in animal social network studies, especially as the field moves towards longitudinal analysis. It also helps researchers wishing to test the power of specific analyses or sampling designs in their own study systems.

## AUTHOR CONTRIBUTIONS

**Matthew J. Silk:** Conceptualization (equal); formal analysis (lead); funding acquisition (lead); investigation (lead); methodology (equal); project administration (equal). **Olivier Gimenez:** Conceptualization (equal); formal analysis (supporting); funding acquisition (supporting); investigation (supporting); methodology (equal); project administration (equal).

## CONFLICT OF INTEREST STATEMENT

The authors have no conflicts of interest to declare.

## Supporting information


Appendix S1

Appendix S2

Appendix S3

## Data Availability

Data and code for the case studies are available at https://github.com/matthewsilk/NETDEM and the R package is available at https://github.com/NETDEM‐project/genNetDem. An archived version of the R package associated with this publication is available at DOI: https://zenodo.org/record/7657423. An archived version of the datasets and code for the case studies is available at https://doi.org/10.5061/dryad.m0cfxpp7s. Note that the case studies used an initial version of the R package available at https://github.com/matthewsilk/NETDEM/genNetDem.
