# Supplementary material for: Generation and applications of simulated datasets to integrate social network and demographic analyses
Source: Ecol Evol. 2023 May 15;13(5):e9871. doi: 10.1002/ece3.9871 (PMC10185435; doi:10.1002/ece3.9871)
Supplement: Supplementary file 1 — Appendix S1 [file ECE3-13-e9871-s003.docx]

**Generation and applications of simulated datasets to integrate social network and demographic analyses: Supplementary Materials 1**

Matthew J Silk^1^* and Olivier Gimenez^1^

1 CEFE, Univ Montpellier, CNRS, EPHE, IRD, Montpellier, France

*corresponding author: [matthewsilk@outlook.com](mailto:matthewsilk@outlook.com)

**Case study workflows**

**
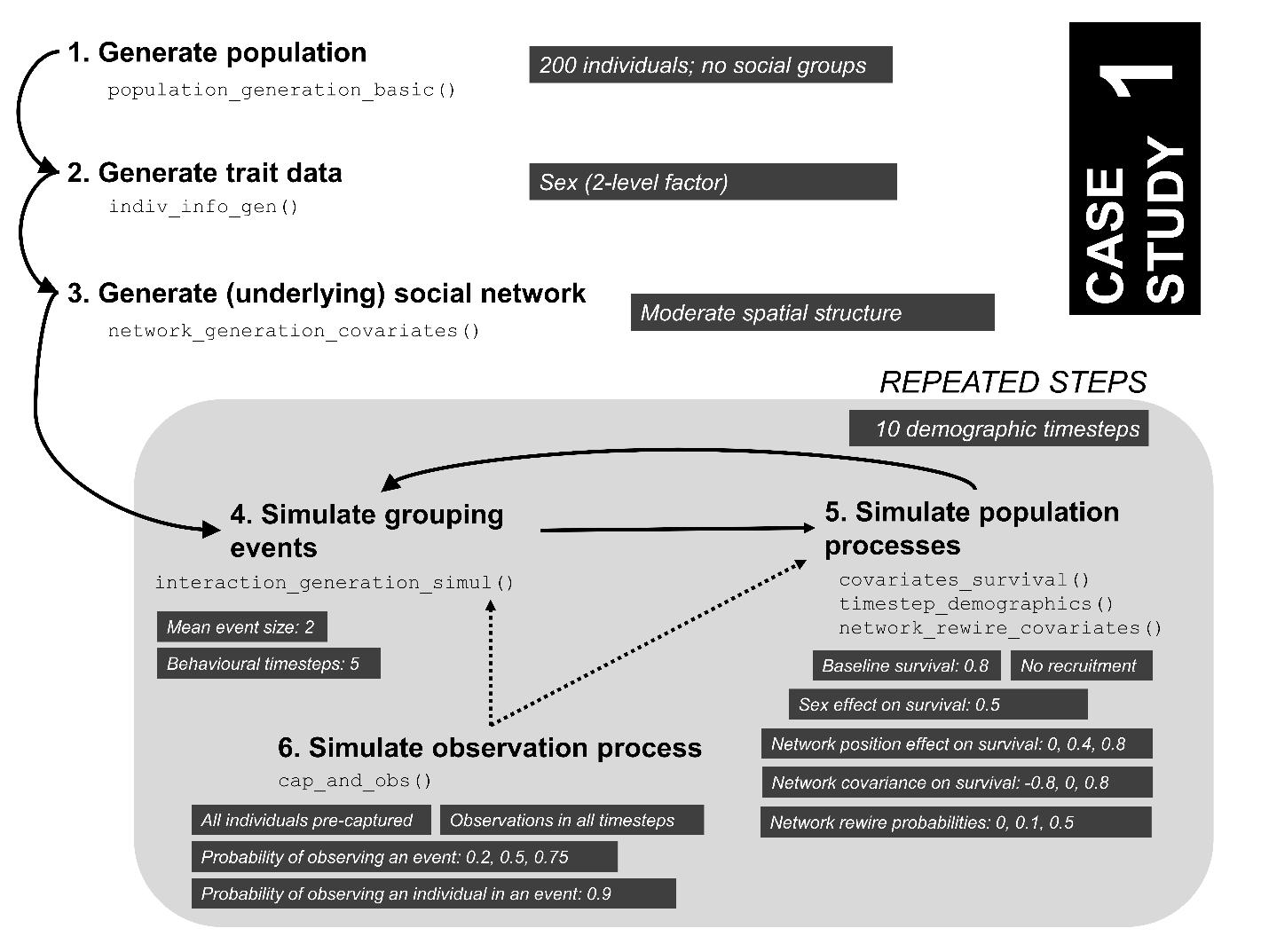
**

Figure S1. Workflow including parameter choices for Case Study 1.


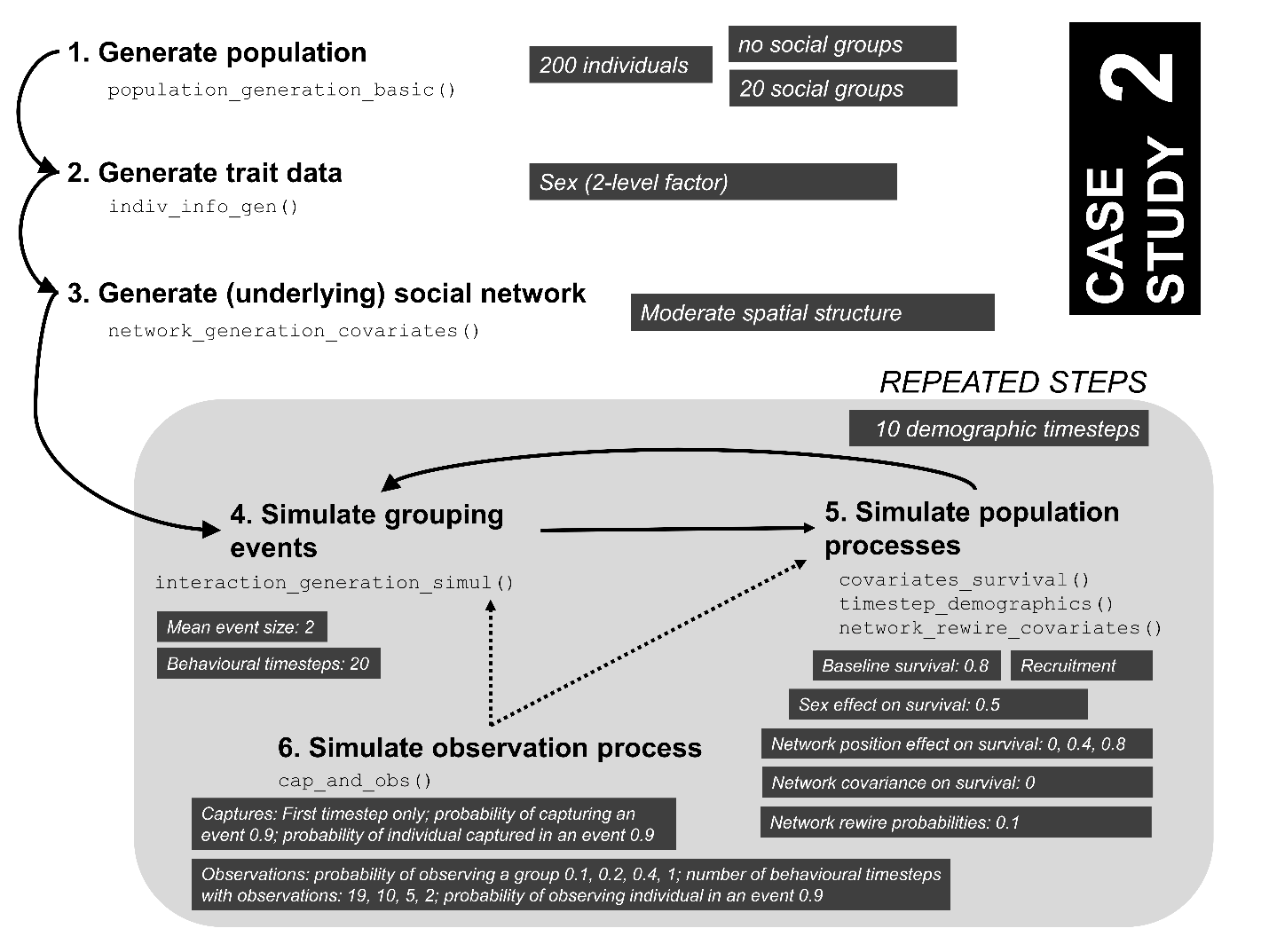


Figure S2. Workflow including parameter choices for Case Study 2.

**Supplementary Results**

**Case Study 1**

Table S1. Full version of Table 1 from the main text. Proportion of simulation runs where 0 falls outside the 89% HDI for different parameter combinations. M1: partial network - cross-sectional imputation; M2: partial network – longitudinal imputation; M3: full network – cross-sectional imputation; M4: full network – longitudinal imputation.

| **Network measure** | **True effect** | **Model** | **Group capture probability** | **Detection rate** |
| --- | --- | --- | --- | --- |
| Strength | 0.0 | M1 | 0.25 | 0.07 |
| Strength | 0.0 | M1 | 0.50 | 0.04 |
| Strength | 0.0 | M1 | 0.75 | 0.05 |
| Strength | 0.0 | M2 | 0.25 | 0.04 |
| Strength | 0.0 | M2 | 0.50 | 0.04 |
| Strength | 0.0 | M2 | 0.75 | 0.06 |
| Strength | 0.0 | M3 | 0.25 | 0.03 |
| Strength | 0.0 | M3 | 0.50 | 0.07 |
| Strength | 0.0 | M3 | 0.75 | 0.04 |
| Strength | 0.0 | M4 | 0.25 | 0.01 |
| Strength | 0.0 | M4 | 0.50 | 0.06 |
| Strength | 0.0 | M4 | 0.75 | 0.05 |
| Betweenness | 0.0 | M1 | 0.25 | 0.06 |
| Betweenness | 0.0 | M1 | 0.50 | 0.04 |
| Betweenness | 0.0 | M1 | 0.75 | 0.05 |
| Betweenness | 0.0 | M2 | 0.25 | 0.06 |
| Betweenness | 0.0 | M2 | 0.50 | 0.06 |
| Betweenness | 0.0 | M2 | 0.75 | 0.03 |
| Betweenness | 0.0 | M3 | 0.25 | 0.10 |
| Betweenness | 0.0 | M3 | 0.50 | 0.03 |
| Betweenness | 0.0 | M3 | 0.75 | 0.06 |
| Betweenness | 0.0 | M4 | 0.25 | 0.09 |
| Betweenness | 0.0 | M4 | 0.50 | 0.04 |
| Betweenness | 0.0 | M4 | 0.75 | 0.07 |
| Strength | 0.4 | M1 | 0.25 | 0.36 |
| Strength | 0.4 | M1 | 0.50 | 0.75 |
| Strength | 0.4 | M1 | 0.75 | 0.88 |
| Strength | 0.4 | M2 | 0.25 | 0.26 |
| Strength | 0.4 | M2 | 0.50 | 0.72 |
| Strength | 0.4 | M2 | 0.75 | 0.89 |
| Strength | 0.4 | M3 | 0.25 | 0.74 |
| Strength | 0.4 | M3 | 0.50 | 0.98 |
| Strength | 0.4 | M3 | 0.75 | 0.99 |
| Strength | 0.4 | M4 | 0.25 | 0.68 |
| Strength | 0.4 | M4 | 0.50 | 0.98 |
| Strength | 0.4 | M4 | 0.75 | 0.99 |
| Betweenness | 0.4 | M1 | 0.25 | 0.10 |
| Betweenness | 0.4 | M1 | 0.50 | 0.26 |
| Betweenness | 0.4 | M1 | 0.75 | 0.63 |
| Betweenness | 0.4 | M2 | 0.25 | 0.11 |
| Betweenness | 0.4 | M2 | 0.50 | 0.25 |
| Betweenness | 0.4 | M2 | 0.75 | 0.64 |
| Betweenness | 0.4 | M3 | 0.25 | 0.71 |
| Betweenness | 0.4 | M3 | 0.50 | 0.94 |
| Betweenness | 0.4 | M3 | 0.75 | 0.99 |
| Betweenness | 0.4 | M4 | 0.25 | 0.65 |
| Betweenness | 0.4 | M4 | 0.50 | 0.94 |
| Betweenness | 0.4 | M4 | 0.75 | 0.99 |
| Strength | 0.8 | M1 | 0.25 | 0.75 |
| Strength | 0.8 | M1 | 0.50 | 0.99 |
| Strength | 0.8 | M1 | 0.75 | 1.00 |
| Strength | 0.8 | M2 | 0.25 | 0.69 |
| Strength | 0.8 | M2 | 0.50 | 0.99 |
| Strength | 0.8 | M2 | 0.75 | 1.00 |
| Strength | 0.8 | M3 | 0.25 | 0.99 |
| Strength | 0.8 | M3 | 0.50 | 1.00 |
| Strength | 0.8 | M3 | 0.75 | 1.00 |
| Strength | 0.8 | M4 | 0.25 | 0.99 |
| Strength | 0.8 | M4 | 0.50 | 1.00 |
| Strength | 0.8 | M4 | 0.75 | 1.00 |
| Betweenness | 0.8 | M1 | 0.25 | 0.16 |
| Betweenness | 0.8 | M1 | 0.50 | 0.70 |
| Betweenness | 0.8 | M1 | 0.75 | 0.99 |
| Betweenness | 0.8 | M2 | 0.25 | 0.18 |
| Betweenness | 0.8 | M2 | 0.50 | 0.68 |
| Betweenness | 0.8 | M2 | 0.75 | 0.99 |
| Betweenness | 0.8 | M3 | 0.25 | 1.00 |
| Betweenness | 0.8 | M3 | 0.50 | 1.00 |
| Betweenness | 0.8 | M3 | 0.75 | 1.00 |
| Betweenness | 0.8 | M4 | 0.25 | 0.98 |
| Betweenness | 0.8 | M4 | 0.50 | 1.00 |
| Betweenness | 0.8 | M4 | 0.75 | 1.00 |

Table S2. Extended version of Table 2 from the main text. Convergence rates of models using different imputation approaches for various parameter combinations. M1: partial network - cross-sectional imputation; M2: partial network – longitudinal imputation; M3: full network – cross-sectional imputation; M4: full network – longitudinal imputation.

| **Network measure** | **True effect** | **Model** | **Group capture probability** | **Convergence rate** |
| --- | --- | --- | --- | --- |
| Strength | 0.0 | M1 | 0.25 | 0.98 |
| Strength | 0.0 | M1 | 0.50 | 0.98 |
| Strength | 0.0 | M1 | 0.75 | 1.00 |
| Strength | 0.0 | M2 | 0.25 | 1.00 |
| Strength | 0.0 | M2 | 0.50 | 1.00 |
| Strength | 0.0 | M2 | 0.75 | 1.00 |
| Strength | 0.0 | M3 | 0.25 | 1.00 |
| Strength | 0.0 | M3 | 0.50 | 1.00 |
| Strength | 0.0 | M3 | 0.75 | 1.00 |
| Strength | 0.0 | M4 | 0.25 | 1.00 |
| Strength | 0.0 | M4 | 0.50 | 1.00 |
| Strength | 0.0 | M4 | 0.75 | 1.00 |
| Betweenness | 0.0 | M1 | 0.25 | 0.70 |
| Betweenness | 0.0 | M1 | 0.50 | 0.94 |
| Betweenness | 0.0 | M1 | 0.75 | 0.99 |
| Betweenness | 0.0 | M2 | 0.25 | 0.97 |
| Betweenness | 0.0 | M2 | 0.50 | 0.99 |
| Betweenness | 0.0 | M2 | 0.75 | 1.00 |
| Betweenness | 0.0 | M3 | 0.25 | 0.97 |
| Betweenness | 0.0 | M3 | 0.50 | 0.98 |
| Betweenness | 0.0 | M3 | 0.75 | 0.97 |
| Betweenness | 0.0 | M4 | 0.25 | 1.00 |
| Betweenness | 0.0 | M4 | 0.50 | 1.00 |
| Betweenness | 0.0 | M4 | 0.75 | 0.99 |
| Strength | 0.4 | M1 | 0.25 | 0.97 |
| Strength | 0.4 | M1 | 0.50 | 0.99 |
| Strength | 0.4 | M1 | 0.75 | 1.00 |
| Strength | 0.4 | M2 | 0.25 | 0.99 |
| Strength | 0.4 | M2 | 0.50 | 1.00 |
| Strength | 0.4 | M2 | 0.75 | 1.00 |
| Strength | 0.4 | M3 | 0.25 | 0.99 |
| Strength | 0.4 | M3 | 0.50 | 1.00 |
| Strength | 0.4 | M3 | 0.75 | 0.99 |
| Strength | 0.4 | M4 | 0.25 | 1.00 |
| Strength | 0.4 | M4 | 0.50 | 1.00 |
| Strength | 0.4 | M4 | 0.75 | 1.00 |
| Betweenness | 0.4 | M1 | 0.25 | 0.62 |
| Betweenness | 0.4 | M1 | 0.50 | 0.95 |
| Betweenness | 0.4 | M1 | 0.75 | 0.98 |
| Betweenness | 0.4 | M2 | 0.25 | 0.97 |
| Betweenness | 0.4 | M2 | 0.50 | 1.00 |
| Betweenness | 0.4 | M2 | 0.75 | 1.00 |
| Betweenness | 0.4 | M3 | 0.25 | 0.97 |
| Betweenness | 0.4 | M3 | 0.50 | 0.99 |
| Betweenness | 0.4 | M3 | 0.75 | 0.99 |
| Betweenness | 0.4 | M4 | 0.25 | 0.99 |
| Betweenness | 0.4 | M4 | 0.50 | 0.99 |
| Betweenness | 0.4 | M4 | 0.75 | 1.00 |
| Strength | 0.8 | M1 | 0.25 | 0.98 |
| Strength | 0.8 | M1 | 0.50 | 1.00 |
| Strength | 0.8 | M1 | 0.75 | 0.99 |
| Strength | 0.8 | M2 | 0.25 | 0.99 |
| Strength | 0.8 | M2 | 0.50 | 1.00 |
| Strength | 0.8 | M2 | 0.75 | 1.00 |
| Strength | 0.8 | M3 | 0.25 | 1.00 |
| Strength | 0.8 | M3 | 0.50 | 1.00 |
| Strength | 0.8 | M3 | 0.75 | 1.00 |
| Strength | 0.8 | M4 | 0.25 | 1.00 |
| Strength | 0.8 | M4 | 0.50 | 1.00 |
| Strength | 0.8 | M4 | 0.75 | 1.00 |
| Betweenness | 0.8 | M1 | 0.25 | 0.63 |
| Betweenness | 0.8 | M1 | 0.50 | 0.92 |
| Betweenness | 0.8 | M1 | 0.75 | 0.98 |
| Betweenness | 0.8 | M2 | 0.25 | 0.94 |
| Betweenness | 0.8 | M2 | 0.50 | 0.99 |
| Betweenness | 0.8 | M2 | 0.75 | 1.00 |
| Betweenness | 0.8 | M3 | 0.25 | 0.96 |
| Betweenness | 0.8 | M3 | 0.50 | 0.95 |
| Betweenness | 0.8 | M3 | 0.75 | 1.00 |
| Betweenness | 0.8 | M4 | 0.25 | 1.00 |
| Betweenness | 0.8 | M4 | 0.50 | 1.00 |
| Betweenness | 0.8 | M4 | 0.75 | 1.00 |

Table S3. Proportion of simulation runs where 0 falls outside the 89% HDI for different parameter combinations focused on the effect of network dynamics when most groups are sampled (group capture probability = 0.75). M1: partial network - cross-sectional imputation; M2: partial network – longitudinal imputation; M3: full network – cross-sectional imputation; M4: full network – longitudinal imputation.

| **Network measure** | **True effect** | **Model** | **Rewiring probability** | **Detection rate** |
| --- | --- | --- | --- | --- |
| Strength | 0.0 | M1 | 0.0 | 0.05 |
| Strength | 0.0 | M1 | 0.1 | 0.03 |
| Strength | 0.0 | M1 | 0.5 | 0.07 |
| Strength | 0.0 | M2 | 0.0 | 0.07 |
| Strength | 0.0 | M2 | 0.1 | 0.03 |
| Strength | 0.0 | M2 | 0.5 | 0.07 |
| Strength | 0.0 | M3 | 0.0 | 0.07 |
| Strength | 0.0 | M3 | 0.1 | 0.05 |
| Strength | 0.0 | M3 | 0.5 | 0.02 |
| Strength | 0.0 | M4 | 0.0 | 0.07 |
| Strength | 0.0 | M4 | 0.1 | 0.05 |
| Strength | 0.0 | M4 | 0.5 | 0.03 |
| Betweenness | 0.0 | M1 | 0.0 | 0.10 |
| Betweenness | 0.0 | M1 | 0.1 | 0.00 |
| Betweenness | 0.0 | M1 | 0.5 | 0.05 |
| Betweenness | 0.0 | M2 | 0.0 | 0.08 |
| Betweenness | 0.0 | M2 | 0.1 | 0.00 |
| Betweenness | 0.0 | M2 | 0.5 | 0.02 |
| Betweenness | 0.0 | M3 | 0.0 | 0.07 |
| Betweenness | 0.0 | M3 | 0.1 | 0.02 |
| Betweenness | 0.0 | M3 | 0.5 | 0.11 |
| Betweenness | 0.0 | M4 | 0.0 | 0.07 |
| Betweenness | 0.0 | M4 | 0.1 | 0.02 |
| Betweenness | 0.0 | M4 | 0.5 | 0.13 |
| Strength | 0.4 | M1 | 0.0 | 0.92 |
| Strength | 0.4 | M1 | 0.1 | 0.87 |
| Strength | 0.4 | M1 | 0.5 | 0.87 |
| Strength | 0.4 | M2 | 0.0 | 0.92 |
| Strength | 0.4 | M2 | 0.1 | 0.87 |
| Strength | 0.4 | M2 | 0.5 | 0.88 |
| Strength | 0.4 | M3 | 0.0 | 1.00 |
| Strength | 0.4 | M3 | 0.1 | 1.00 |
| Strength | 0.4 | M3 | 0.5 | 0.97 |
| Strength | 0.4 | M4 | 0.0 | 1.00 |
| Strength | 0.4 | M4 | 0.1 | 1.00 |
| Strength | 0.4 | M4 | 0.5 | 0.97 |
| Betweenness | 0.4 | M1 | 0.0 | 0.69 |
| Betweenness | 0.4 | M1 | 0.1 | 0.59 |
| Betweenness | 0.4 | M1 | 0.5 | 0.62 |
| Betweenness | 0.4 | M2 | 0.0 | 0.63 |
| Betweenness | 0.4 | M2 | 0.1 | 0.62 |
| Betweenness | 0.4 | M2 | 0.5 | 0.67 |
| Betweenness | 0.4 | M3 | 0.0 | 0.98 |
| Betweenness | 0.4 | M3 | 0.1 | 1.00 |
| Betweenness | 0.4 | M3 | 0.5 | 1.00 |
| Betweenness | 0.4 | M4 | 0.0 | 1.00 |
| Betweenness | 0.4 | M4 | 0.1 | 1.00 |
| Betweenness | 0.4 | M4 | 0.5 | 0.98 |
| Strength | 0.8 | M1 | 0.0 | 1.00 |
| Strength | 0.8 | M1 | 0.1 | 1.00 |
| Strength | 0.8 | M1 | 0.5 | 1.00 |
| Strength | 0.8 | M2 | 0.0 | 1.00 |
| Strength | 0.8 | M2 | 0.1 | 1.00 |
| Strength | 0.8 | M2 | 0.5 | 1.00 |
| Strength | 0.8 | M3 | 0.0 | 1.00 |
| Strength | 0.8 | M3 | 0.1 | 1.00 |
| Strength | 0.8 | M3 | 0.5 | 1.00 |
| Strength | 0.8 | M4 | 0.0 | 1.00 |
| Strength | 0.8 | M4 | 0.1 | 1.00 |
| Strength | 0.8 | M4 | 0.5 | 1.00 |
| Betweenness | 0.8 | M1 | 0.0 | 0.98 |
| Betweenness | 0.8 | M1 | 0.1 | 1.00 |
| Betweenness | 0.8 | M1 | 0.5 | 0.98 |
| Betweenness | 0.8 | M2 | 0.0 | 0.98 |
| Betweenness | 0.8 | M2 | 0.1 | 1.00 |
| Betweenness | 0.8 | M2 | 0.5 | 0.98 |
| Betweenness | 0.8 | M3 | 0.0 | 1.00 |
| Betweenness | 0.8 | M3 | 0.1 | 1.00 |
| Betweenness | 0.8 | M3 | 0.5 | 1.00 |
| Betweenness | 0.8 | M4 | 0.0 | 1.00 |
| Betweenness | 0.8 | M4 | 0.1 | 1.00 |
| Betweenness | 0.8 | M4 | 0.5 | 1.00 |

Table S4. Proportion of simulation runs where 0 falls outside the 89% HDI for different parameter combinations focused on the effect of network dynamics when few groups are sampled (group capture probability = 0.25). M1: partial network - cross-sectional imputation; M2: partial network – longitudinal imputation; M3: full network – cross-sectional imputation; M4: full network – longitudinal imputation.

| **Network measure** | **True effect** | **Model** | **Rewiring probability** | **Detection rate** |
| --- | --- | --- | --- | --- |
| Strength | 0.0 | M1 | 0.0 | 0.10 |
| Strength | 0.0 | M1 | 0.1 | 0.05 |
| Strength | 0.0 | M1 | 0.5 | 0.07 |
| Strength | 0.0 | M2 | 0.0 | 0.08 |
| Strength | 0.0 | M2 | 0.1 | 0.02 |
| Strength | 0.0 | M2 | 0.5 | 0.02 |
| Strength | 0.0 | M3 | 0.0 | 0.05 |
| Strength | 0.0 | M3 | 0.1 | 0.00 |
| Strength | 0.0 | M3 | 0.5 | 0.05 |
| Strength | 0.0 | M4 | 0.0 | 0.00 |
| Strength | 0.0 | M4 | 0.1 | 0.02 |
| Strength | 0.0 | M4 | 0.5 | 0.02 |
| Betweenness | 0.0 | M1 | 0.0 | 0.08 |
| Betweenness | 0.0 | M1 | 0.1 | 0.05 |
| Betweenness | 0.0 | M1 | 0.5 | 0.06 |
| Betweenness | 0.0 | M2 | 0.0 | 0.07 |
| Betweenness | 0.0 | M2 | 0.1 | 0.09 |
| Betweenness | 0.0 | M2 | 0.5 | 0.03 |
| Betweenness | 0.0 | M3 | 0.0 | 0.09 |
| Betweenness | 0.0 | M3 | 0.1 | 0.08 |
| Betweenness | 0.0 | M3 | 0.5 | 0.12 |
| Betweenness | 0.0 | M4 | 0.0 | 0.08 |
| Betweenness | 0.0 | M4 | 0.1 | 0.07 |
| Betweenness | 0.0 | M4 | 0.5 | 0.12 |
| Strength | 0.4 | M1 | 0.0 | 0.27 |
| Strength | 0.4 | M1 | 0.1 | 0.44 |
| Strength | 0.4 | M1 | 0.5 | 0.37 |
| Strength | 0.4 | M2 | 0.0 | 0.20 |
| Strength | 0.4 | M2 | 0.1 | 0.32 |
| Strength | 0.4 | M2 | 0.5 | 0.25 |
| Strength | 0.4 | M3 | 0.0 | 0.63 |
| Strength | 0.4 | M3 | 0.1 | 0.83 |
| Strength | 0.4 | M3 | 0.5 | 0.75 |
| Strength | 0.4 | M4 | 0.0 | 0.57 |
| Strength | 0.4 | M4 | 0.1 | 0.75 |
| Strength | 0.4 | M4 | 0.5 | 0.72 |
| Betweenness | 0.4 | M1 | 0.0 | 0.10 |
| Betweenness | 0.4 | M1 | 0.1 | 0.11 |
| Betweenness | 0.4 | M1 | 0.5 | 0.08 |
| Betweenness | 0.4 | M2 | 0.0 | 0.10 |
| Betweenness | 0.4 | M2 | 0.1 | 0.16 |
| Betweenness | 0.4 | M2 | 0.5 | 0.09 |
| Betweenness | 0.4 | M3 | 0.0 | 0.67 |
| Betweenness | 0.4 | M3 | 0.1 | 0.70 |
| Betweenness | 0.4 | M3 | 0.5 | 0.78 |
| Betweenness | 0.4 | M4 | 0.0 | 0.61 |
| Betweenness | 0.4 | M4 | 0.1 | 0.62 |
| Betweenness | 0.4 | M4 | 0.5 | 0.73 |
| Strength | 0.8 | M1 | 0.0 | 0.75 |
| Strength | 0.8 | M1 | 0.1 | 0.76 |
| Strength | 0.8 | M1 | 0.5 | 0.74 |
| Strength | 0.8 | M2 | 0.0 | 0.71 |
| Strength | 0.8 | M2 | 0.1 | 0.68 |
| Strength | 0.8 | M2 | 0.5 | 0.67 |
| Strength | 0.8 | M3 | 0.0 | 1.00 |
| Strength | 0.8 | M3 | 0.1 | 0.98 |
| Strength | 0.8 | M3 | 0.5 | 1.00 |
| Strength | 0.8 | M4 | 0.0 | 1.00 |
| Strength | 0.8 | M4 | 0.1 | 1.00 |
| Strength | 0.8 | M4 | 0.5 | 0.97 |
| Betweenness | 0.8 | M1 | 0.0 | 0.11 |
| Betweenness | 0.8 | M1 | 0.1 | 0.23 |
| Betweenness | 0.8 | M1 | 0.5 | 0.12 |
| Betweenness | 0.8 | M2 | 0.0 | 0.15 |
| Betweenness | 0.8 | M2 | 0.1 | 0.28 |
| Betweenness | 0.8 | M2 | 0.5 | 0.12 |
| Betweenness | 0.8 | M3 | 0.0 | 1.00 |
| Betweenness | 0.8 | M3 | 0.1 | 1.00 |
| Betweenness | 0.8 | M3 | 0.5 | 1.00 |
| Betweenness | 0.8 | M4 | 0.0 | 1.00 |
| Betweenness | 0.8 | M4 | 0.1 | 0.98 |
| Betweenness | 0.8 | M4 | 0.5 | 0.97 |

Table S5. Proportion of simulation runs where 0 falls outside the 89% HDI for different parameter combinations focused on the effect of network covariance when most groups are sampled (group capture probability = 0.75). M1: partial network - cross-sectional imputation; M2: partial network – longitudinal imputation; M3: full network – cross-sectional imputation; M4: full network – longitudinal imputation.

| **Network measure** | **True effect** | **Model** | **Network covariance** | **Detection rate** |
| --- | --- | --- | --- | --- |
| Strength | 0.0 | M1 | -0.8 | 0.02 |
| Strength | 0.0 | M1 | 0.0 | 0.05 |
| Strength | 0.0 | M1 | 0.8 | 0.08 |
| Strength | 0.0 | M2 | -0.8 | 0.03 |
| Strength | 0.0 | M2 | 0.0 | 0.03 |
| Strength | 0.0 | M2 | 0.8 | 0.10 |
| Strength | 0.0 | M3 | -0.8 | 0.05 |
| Strength | 0.0 | M3 | 0.0 | 0.03 |
| Strength | 0.0 | M3 | 0.8 | 0.05 |
| Strength | 0.0 | M4 | -0.8 | 0.03 |
| Strength | 0.0 | M4 | 0.0 | 0.07 |
| Strength | 0.0 | M4 | 0.8 | 0.05 |
| Betweenness | 0.0 | M1 | -0.8 | 0.08 |
| Betweenness | 0.0 | M1 | 0.0 | 0.02 |
| Betweenness | 0.0 | M1 | 0.8 | 0.05 |
| Betweenness | 0.0 | M2 | -0.8 | 0.07 |
| Betweenness | 0.0 | M2 | 0.0 | 0.02 |
| Betweenness | 0.0 | M2 | 0.8 | 0.02 |
| Betweenness | 0.0 | M3 | -0.8 | 0.08 |
| Betweenness | 0.0 | M3 | 0.0 | 0.04 |
| Betweenness | 0.0 | M3 | 0.8 | 0.07 |
| Betweenness | 0.0 | M4 | -0.8 | 0.10 |
| Betweenness | 0.0 | M4 | 0.0 | 0.05 |
| Betweenness | 0.0 | M4 | 0.8 | 0.07 |
| Strength | 0.4 | M1 | -0.8 | 0.82 |
| Strength | 0.4 | M1 | 0.0 | 0.95 |
| Strength | 0.4 | M1 | 0.8 | 0.88 |
| Strength | 0.4 | M2 | -0.8 | 0.85 |
| Strength | 0.4 | M2 | 0.0 | 0.95 |
| Strength | 0.4 | M2 | 0.8 | 0.87 |
| Strength | 0.4 | M3 | -0.8 | 0.97 |
| Strength | 0.4 | M3 | 0.0 | 1.00 |
| Strength | 0.4 | M3 | 0.8 | 1.00 |
| Strength | 0.4 | M4 | -0.8 | 0.97 |
| Strength | 0.4 | M4 | 0.0 | 1.00 |
| Strength | 0.4 | M4 | 0.8 | 1.00 |
| Betweenness | 0.4 | M1 | -0.8 | 0.68 |
| Betweenness | 0.4 | M1 | 0.0 | 0.64 |
| Betweenness | 0.4 | M1 | 0.8 | 0.57 |
| Betweenness | 0.4 | M2 | -0.8 | 0.67 |
| Betweenness | 0.4 | M2 | 0.0 | 0.68 |
| Betweenness | 0.4 | M2 | 0.8 | 0.57 |
| Betweenness | 0.4 | M3 | -0.8 | 1.00 |
| Betweenness | 0.4 | M3 | 0.0 | 1.00 |
| Betweenness | 0.4 | M3 | 0.8 | 0.98 |
| Betweenness | 0.4 | M4 | -0.8 | 0.98 |
| Betweenness | 0.4 | M4 | 0.0 | 1.00 |
| Betweenness | 0.4 | M4 | 0.8 | 1.00 |
| Strength | 0.8 | M1 | -0.8 | 1.00 |
| Strength | 0.8 | M1 | 0.0 | 1.00 |
| Strength | 0.8 | M1 | 0.8 | 1.00 |
| Strength | 0.8 | M2 | -0.8 | 1.00 |
| Strength | 0.8 | M2 | 0.0 | 1.00 |
| Strength | 0.8 | M2 | 0.8 | 1.00 |
| Strength | 0.8 | M3 | -0.8 | 1.00 |
| Strength | 0.8 | M3 | 0.0 | 1.00 |
| Strength | 0.8 | M3 | 0.8 | 1.00 |
| Strength | 0.8 | M4 | -0.8 | 1.00 |
| Strength | 0.8 | M4 | 0.0 | 1.00 |
| Strength | 0.8 | M4 | 0.8 | 1.00 |
| Betweenness | 0.8 | M1 | -0.8 | 1.00 |
| Betweenness | 0.8 | M1 | 0.0 | 0.98 |
| Betweenness | 0.8 | M1 | 0.8 | 0.98 |
| Betweenness | 0.8 | M2 | -0.8 | 0.98 |
| Betweenness | 0.8 | M2 | 0.0 | 1.00 |
| Betweenness | 0.8 | M2 | 0.8 | 0.98 |
| Betweenness | 0.8 | M3 | -0.8 | 1.00 |
| Betweenness | 0.8 | M3 | 0.0 | 1.00 |
| Betweenness | 0.8 | M3 | 0.8 | 1.00 |
| Betweenness | 0.8 | M4 | -0.8 | 1.00 |
| Betweenness | 0.8 | M4 | 0.0 | 1.00 |
| Betweenness | 0.8 | M4 | 0.8 | 1.00 |

Table S6. Proportion of simulation runs where 0 falls outside the 89% HDI for different parameter combinations focused on the effect of network covariance when few groups are sampled (group capture probability = 0.25). M1: partial network - cross-sectional imputation; M2: partial network – longitudinal imputation; M3: full network – cross-sectional imputation; M4: full network – longitudinal imputation.

| **Network measure** | **True effect** | **Model** | **Network covariance** | **Detection rate** |
| --- | --- | --- | --- | --- |
| Strength | 0.0 | M1 | -0.8 | 0.05 |
| Strength | 0.0 | M1 | 0.0 | 0.07 |
| Strength | 0.0 | M1 | 0.8 | 0.10 |
| Strength | 0.0 | M2 | -0.8 | 0.05 |
| Strength | 0.0 | M2 | 0.0 | 0.00 |
| Strength | 0.0 | M2 | 0.8 | 0.07 |
| Strength | 0.0 | M3 | -0.8 | 0.03 |
| Strength | 0.0 | M3 | 0.0 | 0.02 |
| Strength | 0.0 | M3 | 0.8 | 0.05 |
| Strength | 0.0 | M4 | -0.8 | 0.00 |
| Strength | 0.0 | M4 | 0.0 | 0.03 |
| Strength | 0.0 | M4 | 0.8 | 0.00 |
| Betweenness | 0.0 | M1 | -0.8 | 0.00 |
| Betweenness | 0.0 | M1 | 0.0 | 0.07 |
| Betweenness | 0.0 | M1 | 0.8 | 0.12 |
| Betweenness | 0.0 | M2 | -0.8 | 0.07 |
| Betweenness | 0.0 | M2 | 0.0 | 0.02 |
| Betweenness | 0.0 | M2 | 0.8 | 0.10 |
| Betweenness | 0.0 | M3 | -0.8 | 0.05 |
| Betweenness | 0.0 | M3 | 0.0 | 0.10 |
| Betweenness | 0.0 | M3 | 0.8 | 0.14 |
| Betweenness | 0.0 | M4 | -0.8 | 0.03 |
| Betweenness | 0.0 | M4 | 0.0 | 0.13 |
| Betweenness | 0.0 | M4 | 0.8 | 0.10 |
| Strength | 0.4 | M1 | -0.8 | 0.32 |
| Strength | 0.4 | M1 | 0.0 | 0.40 |
| Strength | 0.4 | M1 | 0.8 | 0.37 |
| Strength | 0.4 | M2 | -0.8 | 0.22 |
| Strength | 0.4 | M2 | 0.0 | 0.28 |
| Strength | 0.4 | M2 | 0.8 | 0.27 |
| Strength | 0.4 | M3 | -0.8 | 0.73 |
| Strength | 0.4 | M3 | 0.0 | 0.73 |
| Strength | 0.4 | M3 | 0.8 | 0.75 |
| Strength | 0.4 | M4 | -0.8 | 0.67 |
| Strength | 0.4 | M4 | 0.0 | 0.70 |
| Strength | 0.4 | M4 | 0.8 | 0.67 |
| Betweenness | 0.4 | M1 | -0.8 | 0.08 |
| Betweenness | 0.4 | M1 | 0.0 | 0.13 |
| Betweenness | 0.4 | M1 | 0.8 | 0.08 |
| Betweenness | 0.4 | M2 | -0.8 | 0.14 |
| Betweenness | 0.4 | M2 | 0.0 | 0.12 |
| Betweenness | 0.4 | M2 | 0.8 | 0.09 |
| Betweenness | 0.4 | M3 | -0.8 | 0.83 |
| Betweenness | 0.4 | M3 | 0.0 | 0.61 |
| Betweenness | 0.4 | M3 | 0.8 | 0.69 |
| Betweenness | 0.4 | M4 | -0.8 | 0.71 |
| Betweenness | 0.4 | M4 | 0.0 | 0.63 |
| Betweenness | 0.4 | M4 | 0.8 | 0.61 |
| Strength | 0.8 | M1 | -0.8 | 0.68 |
| Strength | 0.8 | M1 | 0.0 | 0.78 |
| Strength | 0.8 | M1 | 0.8 | 0.79 |
| Strength | 0.8 | M2 | -0.8 | 0.65 |
| Strength | 0.8 | M2 | 0.0 | 0.72 |
| Strength | 0.8 | M2 | 0.8 | 0.69 |
| Strength | 0.8 | M3 | -0.8 | 1.00 |
| Strength | 0.8 | M3 | 0.0 | 0.98 |
| Strength | 0.8 | M3 | 0.8 | 1.00 |
| Strength | 0.8 | M4 | -0.8 | 0.98 |
| Strength | 0.8 | M4 | 0.0 | 1.00 |
| Strength | 0.8 | M4 | 0.8 | 0.98 |
| Betweenness | 0.8 | M1 | -0.8 | 0.05 |
| Betweenness | 0.8 | M1 | 0.0 | 0.20 |
| Betweenness | 0.8 | M1 | 0.8 | 0.25 |
| Betweenness | 0.8 | M2 | -0.8 | 0.09 |
| Betweenness | 0.8 | M2 | 0.0 | 0.21 |
| Betweenness | 0.8 | M2 | 0.8 | 0.25 |
| Betweenness | 0.8 | M3 | -0.8 | 1.00 |
| Betweenness | 0.8 | M3 | 0.0 | 1.00 |
| Betweenness | 0.8 | M3 | 0.8 | 1.00 |
| Betweenness | 0.8 | M4 | -0.8 | 0.98 |
| Betweenness | 0.8 | M4 | 0.0 | 0.98 |
| Betweenness | 0.8 | M4 | 0.8 | 0.98 |


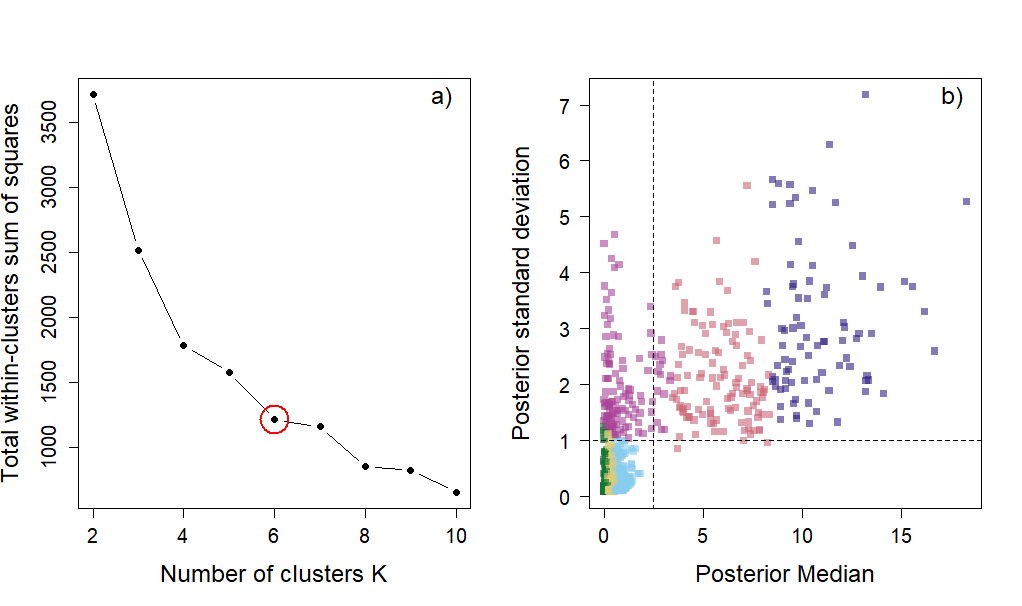


Figure S3. Results from a k-means clustering analysis of posterior medians and standard deviations. a) the elbow method for determining an appropriate number of clusters to use as an outcome of within-cluster sums of squares with six clusters identified with the red circle. b) an illustration of the distribution of the six clusters (point colours represent cluster ID). Models in the blue, yellow and green clusters were assumed to have converged representing 97.5% of model runs.


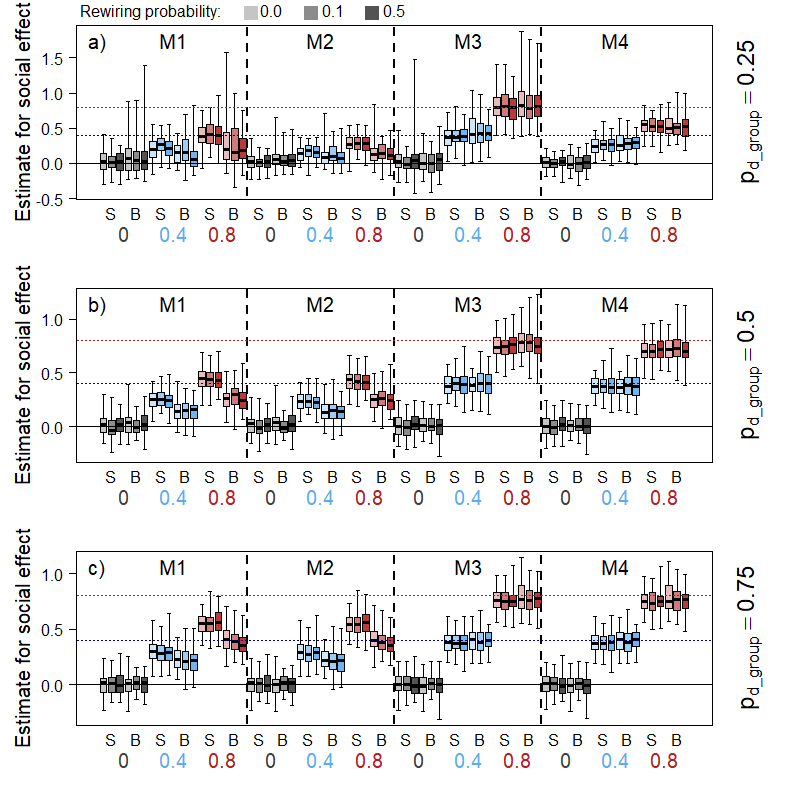


Figure S4. Distribution of posterior medians for the social effect of survival for different combinations of model (M1: partial network - cross-sectional imputation; M2: partial network – longitudinal imputation; M3: full network – cross-sectional imputation; M4: full network – longitudinal imputation), network measure (S=Strength; B=Betweenness), true effect size (box colour) and network dynamics (box shading) when a) 25% of groups are sampled, b) 50% of groups are sampled and c) 75% of groups are sampled. The solid central line represents the median, boxes the interquartile range and whiskers the full range of values.


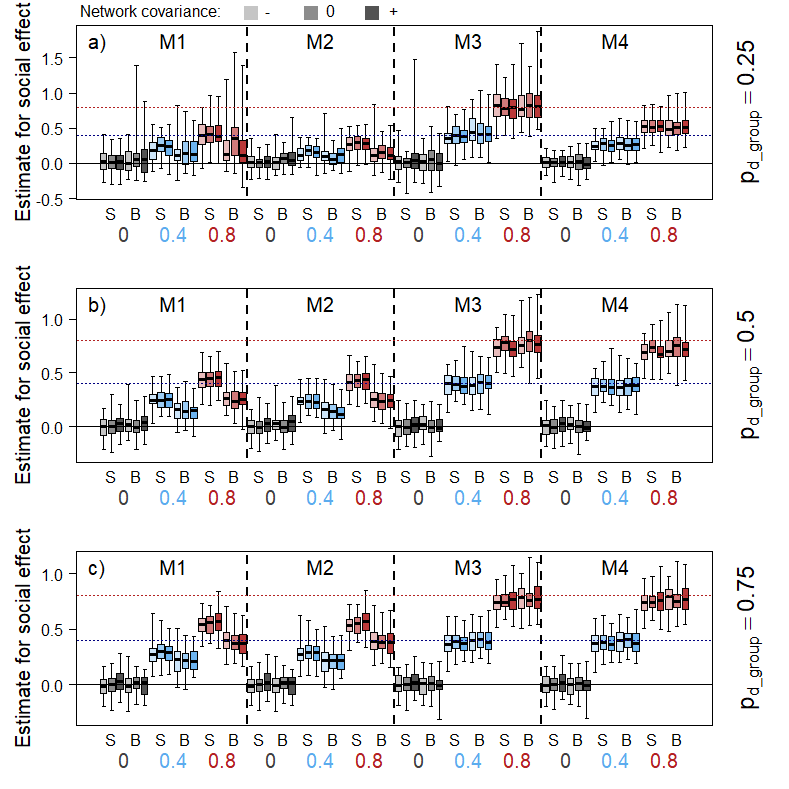


Figure S5. Distribution of posterior medians for the social effect of survival for different combinations of model (M1: partial network - cross-sectional imputation; M2: partial network – longitudinal imputation; M3: full network – cross-sectional imputation; M4: full network – longitudinal imputation), network measure (S=Strength; B=Betweenness), true effect size (box colour) and network covariance (box shading) when a) 25% of groups are sampled, b) 50% of groups are sampled and c) 75% of groups are sampled. The solid central line represents the median, boxes the interquartile range and whiskers the full range of values.


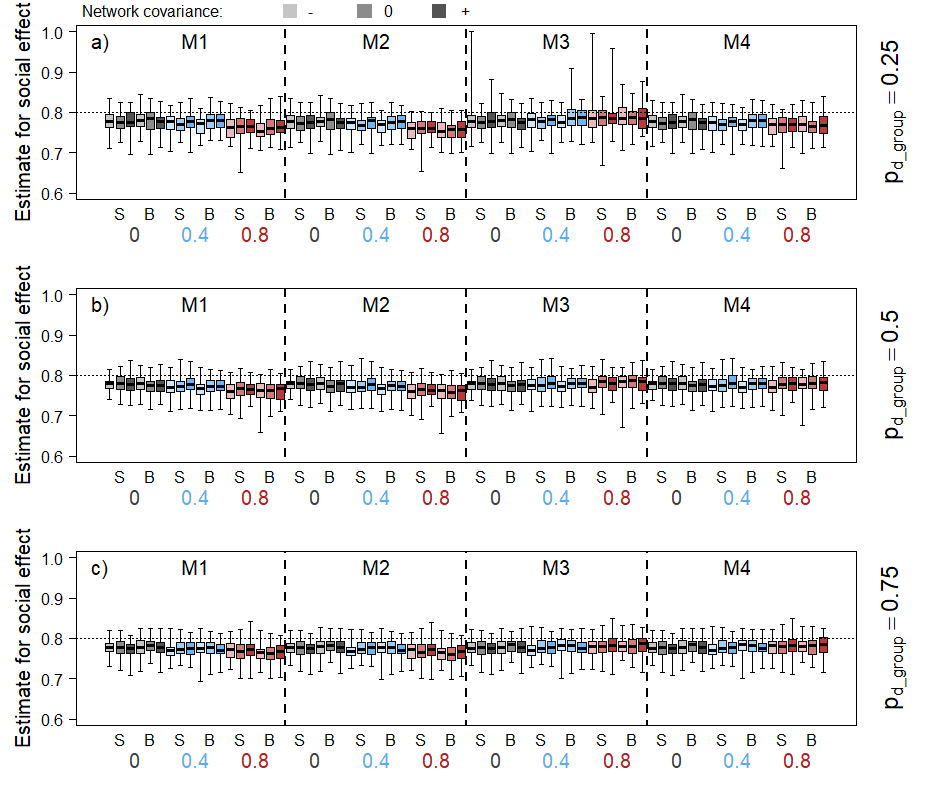
Figure S6. Distribution of posterior medians for the baseline survival probability for different combinations of model (M1: partial network - cross-sectional imputation; M2: partial network – longitudinal imputation; M3: full network – cross-sectional imputation; M4: full network – longitudinal imputation), network measure (S=Strength; B=Betweenness), true effect size (box colour) and network covariance (box shading) when a) 25% of groups are sampled, b) 50% of groups are sampled and c) 75% of groups are sampled. The solid central line represents the median, boxes the interquartile range and whiskers the full range of values.


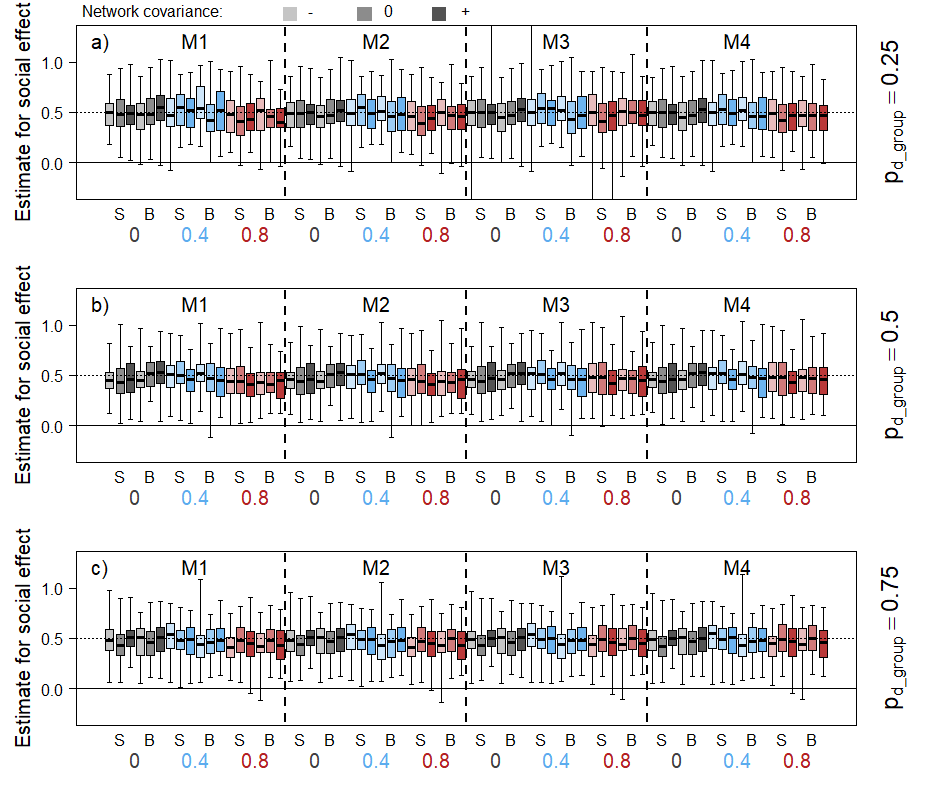


Figure S7. Distribution of posterior medians for the sex effect on survival for different combinations of model (M1: partial network - cross-sectional imputation; M2: partial network – longitudinal imputation; M3: full network – cross-sectional imputation; M4: full network – longitudinal imputation), network measure (S=Strength; B=Betweenness), true effect size (box colour) and network covariance (box shading) when a) 25% of groups are sampled, b) 50% of groups are sampled and c) 75% of groups are sampled. The solid central line represents the median, boxes the interquartile range and whiskers the full range of values.


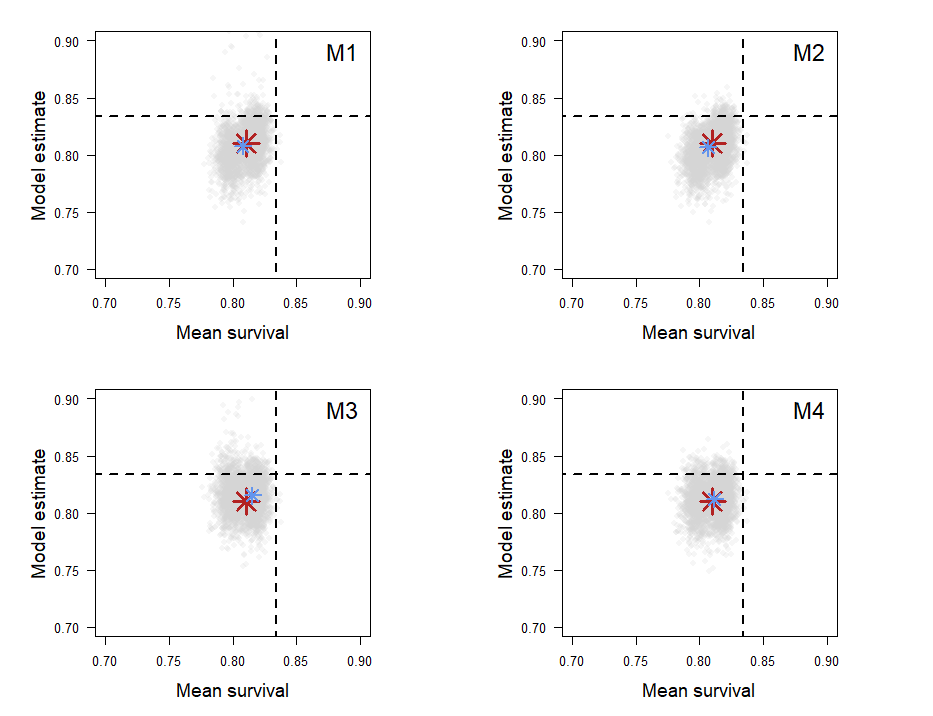


Figure S8. Plot showing mean survival probabilities in the simulations versus model estimated survival probabilities (assuming equal numbers of each sex) for each model (different forms of imputation) from each simulation run. Dotted lines indicate the expected mean based on simulation inputs assuming equal numbers of each sex. While model estimates accurately reflect the mean values of survival generated by the simulations, these are downward-biased from input values.

**Case Study 2**

| **Network measure** | **True effect** | **Social structure** | **Sampling design** | **Detection rate** |
| --- | --- | --- | --- | --- |
| Strength | 0.4 | Communities | 0.1 | 0.35 |
| Strength | 0.4 | No communities | 0.1 | 0.45 |
| Strength | 0.4 | Communities | 0.2 | 0.50 |
| Strength | 0.4 | No communities | 0.2 | 0.75 |
| Strength | 0.4 | Communities | 0.4 | 0.40 |
| Strength | 0.4 | No communities | 0.4 | 0.35 |
| Strength | 0.4 | Communities | 1.0 | 0.55 |
| Strength | 0.4 | No communities | 1.0 | 0.70 |
| Betweenness | 0.4 | Communities | 0.1 | 0.15 |
| Betweenness | 0.4 | No communities | 0.1 | 0.15 |
| Betweenness | 0.4 | Communities | 0.2 | 0.10 |
| Betweenness | 0.4 | No communities | 0.2 | 0.25 |
| Betweenness | 0.4 | Communities | 0.4 | 0.05 |
| Betweenness | 0.4 | No communities | 0.4 | 0.10 |
| Betweenness | 0.4 | Communities | 1.0 | 0.15 |
| Betweenness | 0.4 | No communities | 1.0 | 0.00 |
| Strength | 0.8 | Communities | 0.1 | 0.85 |
| Strength | 0.8 | No communities | 0.1 | 1.00 |
| Strength | 0.8 | Communities | 0.2 | 0.85 |
| Strength | 0.8 | No communities | 0.2 | 0.95 |
| Strength | 0.8 | Communities | 0.4 | 1.00 |
| Strength | 0.8 | No communities | 0.4 | 0.95 |
| Strength | 0.8 | Communities | 1.0 | 1.00 |
| Strength | 0.8 | No communities | 1.0 | 0.95 |
| Betweenness | 0.8 | Communities | 0.1 | 0.20 |
| Betweenness | 0.8 | No communities | 0.1 | 0.05 |
| Betweenness | 0.8 | Communities | 0.2 | 0.25 |
| Betweenness | 0.8 | No communities | 0.2 | 0.35 |
| Betweenness | 0.8 | Communities | 0.4 | 0.25 |
| Betweenness | 0.8 | No communities | 0.4 | 0.35 |
| Betweenness | 0.8 | Communities | 1.0 | 0.10 |
| Betweenness | 0.8 | No communities | 1.0 | 0.25 |

Table S7. Proportion of simulation runs where 0 falls outside the 89% HDI for different parameter combinations with the probability of within-group detection set to 0.75.

Table S8. Proportion of simulation runs where 0 falls outside the 89% HDI for different parameter combinations with the probability of within-group detection set to 0.5.

| **Network measure** | **True effect** | **Social structure** | **Sampling design** | **Detection rate** |
| --- | --- | --- | --- | --- |
| Strength | 0.4 | Communities | 0.1 | 0.40 |
| Strength | 0.4 | No communities | 0.1 | 0.25 |
| Strength | 0.4 | Communities | 0.2 | 0.35 |
| Strength | 0.4 | No communities | 0.2 | 0.55 |
| Strength | 0.4 | Communities | 0.4 | 0.35 |
| Strength | 0.4 | No communities | 0.4 | 0.30 |
| Strength | 0.4 | Communities | 1.0 | 0.35 |
| Strength | 0.4 | No communities | 1.0 | 0.40 |
| Betweenness | 0.4 | Communities | 0.1 | 0.10 |
| Betweenness | 0.4 | No communities | 0.1 | 0.05 |
| Betweenness | 0.4 | Communities | 0.2 | 0.05 |
| Betweenness | 0.4 | No communities | 0.2 | 0.10 |
| Betweenness | 0.4 | Communities | 0.4 | 0.10 |
| Betweenness | 0.4 | No communities | 0.4 | 0.15 |
| Betweenness | 0.4 | Communities | 1.0 | 0.15 |
| Betweenness | 0.4 | No communities | 1.0 | 0.15 |
| Strength | 0.8 | Communities | 0.1 | 0.75 |
| Strength | 0.8 | No communities | 0.1 | 0.90 |
| Strength | 0.8 | Communities | 0.2 | 0.80 |
| Strength | 0.8 | No communities | 0.2 | 0.95 |
| Strength | 0.8 | Communities | 0.4 | 0.80 |
| Strength | 0.8 | No communities | 0.4 | 0.85 |
| Strength | 0.8 | Communities | 1.0 | 1.00 |
| Strength | 0.8 | No communities | 1.0 | 0.85 |
| Betweenness | 0.8 | Communities | 0.1 | 0.20 |
| Betweenness | 0.8 | No communities | 0.1 | 0.20 |
| Betweenness | 0.8 | Communities | 0.2 | 0.30 |
| Betweenness | 0.8 | No communities | 0.2 | 0.15 |
| Betweenness | 0.8 | Communities | 0.4 | 0.00 |
| Betweenness | 0.8 | No communities | 0.4 | 0.40 |
| Betweenness | 0.8 | Communities | 1.0 | 0.25 |
| Betweenness | 0.8 | No communities | 1.0 | 0.20 |

Table S9. Proportion of simulation runs where 0 falls outside the 89% HDI for different parameter combinations when the true effect size is zero.

| **Network measure** | **Social structure** | **Sampling design** | **Within-group detection probability** | **Detection rate** |
| --- | --- | --- | --- | --- |
| Strength | Communities | 0.1 | 0.50 | 0.00 |
| Strength | No communities | 0.1 | 0.50 | 0.05 |
| Strength | Communities | 0.2 | 0.50 | 0.10 |
| Strength | No communities | 0.2 | 0.50 | 0.10 |
| Strength | Communities | 0.4 | 0.50 | 0.10 |
| Strength | No communities | 0.4 | 0.50 | 0.05 |
| Strength | Communities | 1.0 | 0.50 | 0.05 |
| Strength | No communities | 1.0 | 0.50 | 0.10 |
| Betweenness | Communities | 0.1 | 0.50 | 0.00 |
| Betweenness | No communities | 0.1 | 0.50 | 0.05 |
| Betweenness | Communities | 0.2 | 0.50 | 0.05 |
| Betweenness | No communities | 0.2 | 0.50 | 0.00 |
| Betweenness | Communities | 0.4 | 0.50 | 0.10 |
| Betweenness | No communities | 0.4 | 0.50 | 0.05 |
| Betweenness | Communities | 1.0 | 0.50 | 0.10 |
| Betweenness | No communities | 1.0 | 0.50 | 0.10 |
| Strength | Communities | 0.1 | 0.75 | 0.05 |
| Strength | No communities | 0.1 | 0.75 | 0.10 |
| Strength | Communities | 0.2 | 0.75 | 0.00 |
| Strength | No communities | 0.2 | 0.75 | 0.05 |
| Strength | Communities | 0.4 | 0.75 | 0.10 |
| Strength | No communities | 0.4 | 0.75 | 0.10 |
| Strength | Communities | 1.0 | 0.75 | 0.05 |
| Strength | No communities | 1.0 | 0.75 | 0.20 |
| Betweenness | Communities | 0.1 | 0.75 | 0.10 |
| Betweenness | No communities | 0.1 | 0.75 | 0.10 |
| Betweenness | Communities | 0.2 | 0.75 | 0.05 |
| Betweenness | No communities | 0.2 | 0.75 | 0.10 |
| Betweenness | Communities | 0.4 | 0.75 | 0.05 |
| Betweenness | No communities | 0.4 | 0.75 | 0.05 |
| Betweenness | Communities | 1.0 | 0.75 | 0.10 |
| Betweenness | No communities | 1.0 | 0.75 | 0.05 |
| Strength | Communities | 0.1 | 1.00 | 0.00 |
| Strength | No communities | 0.1 | 1.00 | 0.00 |
| Strength | Communities | 0.2 | 1.00 | 0.00 |
| Strength | No communities | 0.2 | 1.00 | 0.05 |
| Strength | Communities | 0.4 | 1.00 | 0.05 |
| Strength | No communities | 0.4 | 1.00 | 0.00 |
| Strength | Communities | 1.0 | 1.00 | 0.00 |
| Strength | No communities | 1.0 | 1.00 | 0.00 |
| Betweenness | Communities | 0.1 | 1.00 | 0.00 |
| Betweenness | No communities | 0.1 | 1.00 | 0.00 |
| Betweenness | Communities | 0.2 | 1.00 | 0.00 |
| Betweenness | No communities | 0.2 | 1.00 | 0.05 |
| Betweenness | Communities | 0.4 | 1.00 | 0.15 |
| Betweenness | No communities | 0.4 | 1.00 | 0.00 |
| Betweenness | Communities | 1.0 | 1.00 | 0.05 |
| Betweenness | No communities | 1.0 | 1.00 | 0.25 |


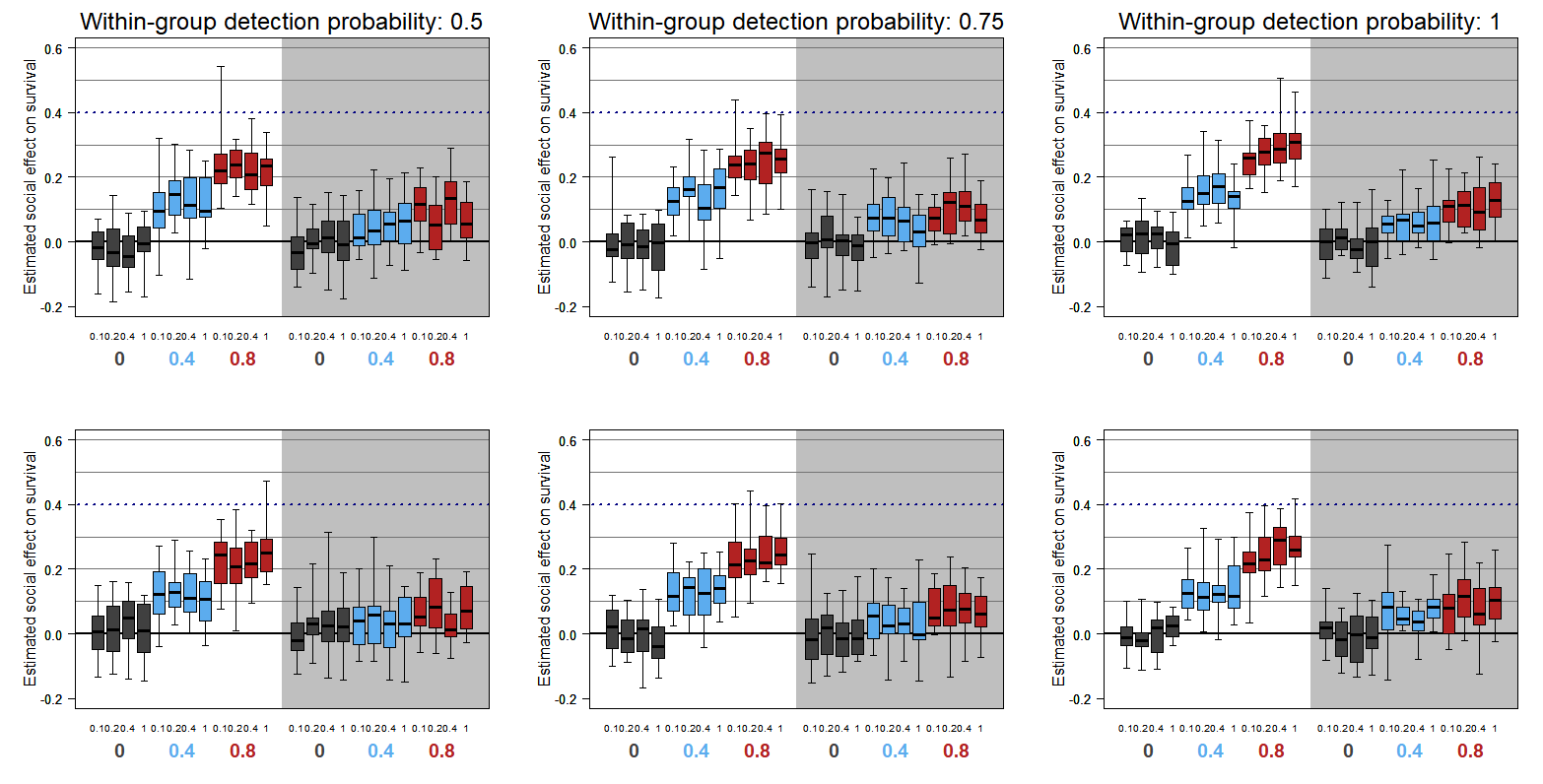


Figure S9. Full version of Figure 5 from the main text. The impacts of sampling design (within-plot: sets of boxes of the same colour), within-group detection probability (columns) and social structure (rows) on Cormack-Jolly-Seber estimates of social effects on survival probability for a range of simulated effect sizes (colours of boxes). Boxplots show the distribution of posterior medians from multiple simulation runs with the solid line the median, boxes the interquartile range and whiskers the full range of values. We illustrate contexts in which a local measure of centrality (strength) and global measure of centrality (betweenness) are used as explanatory variables. The blue-dotted line indicates the accurate parameter estimate when the true effect size is 0.4 (the equivalent line for 0.8 is not illustrated).


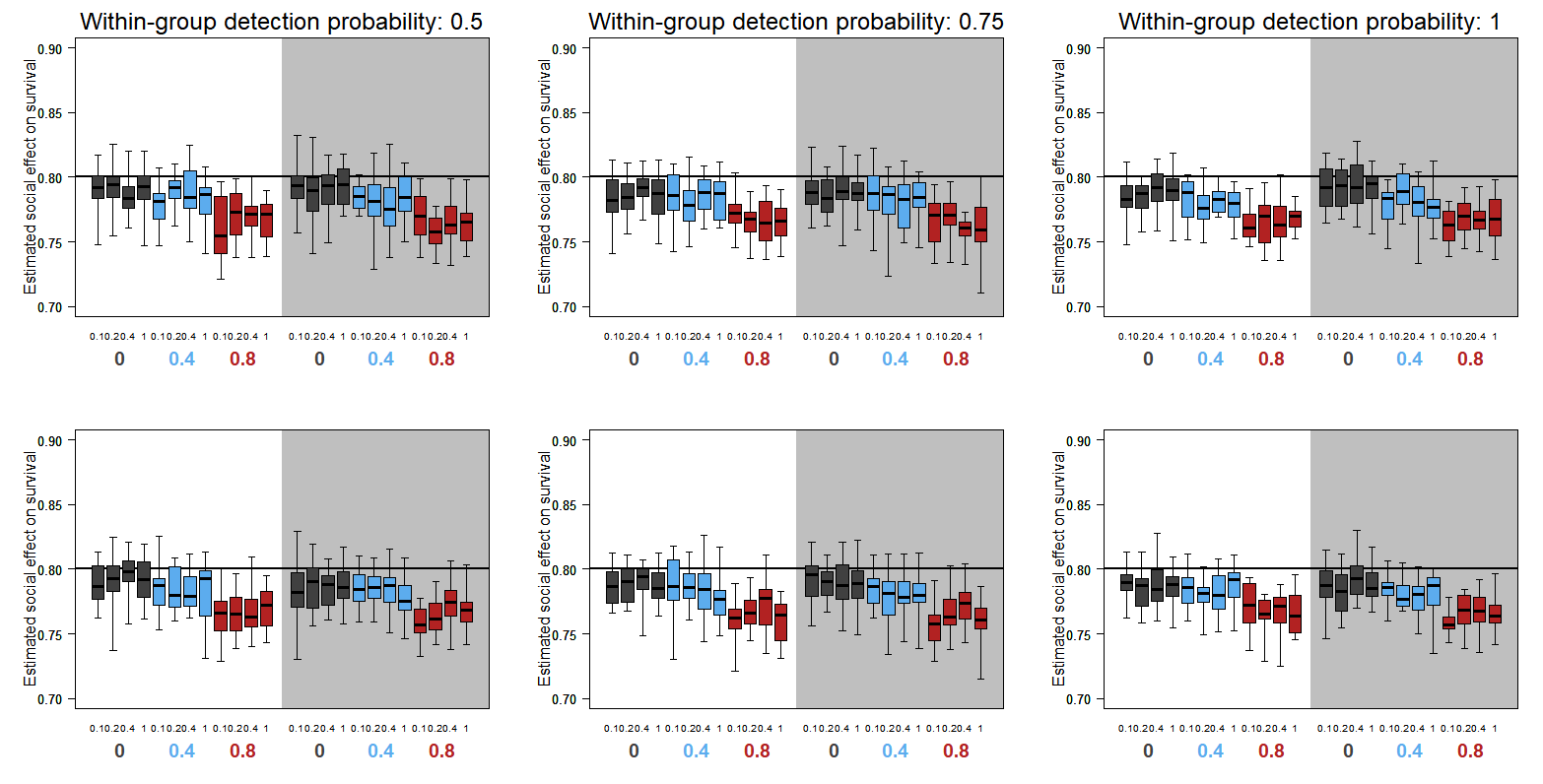


Figure S10. The impacts of sampling design (within-plot: sets of boxes of the same colour), within-group detection probability (columns) and social structure (rows) on Cormack-Jolly-Seber estimates of baseline survival probability for a range of simulated effect sizes (colours of boxes). Boxplots show the distribution of posterior medians from multiple simulation runs with the solid line the median, boxes the interquartile range and whiskers the full range of values. We illustrate contexts in which a local measure of centrality (strength) and global measure of centrality (betweenness) are used as explanatory variables. The black line indicates the value used for simulations (although see also Fig. S9).


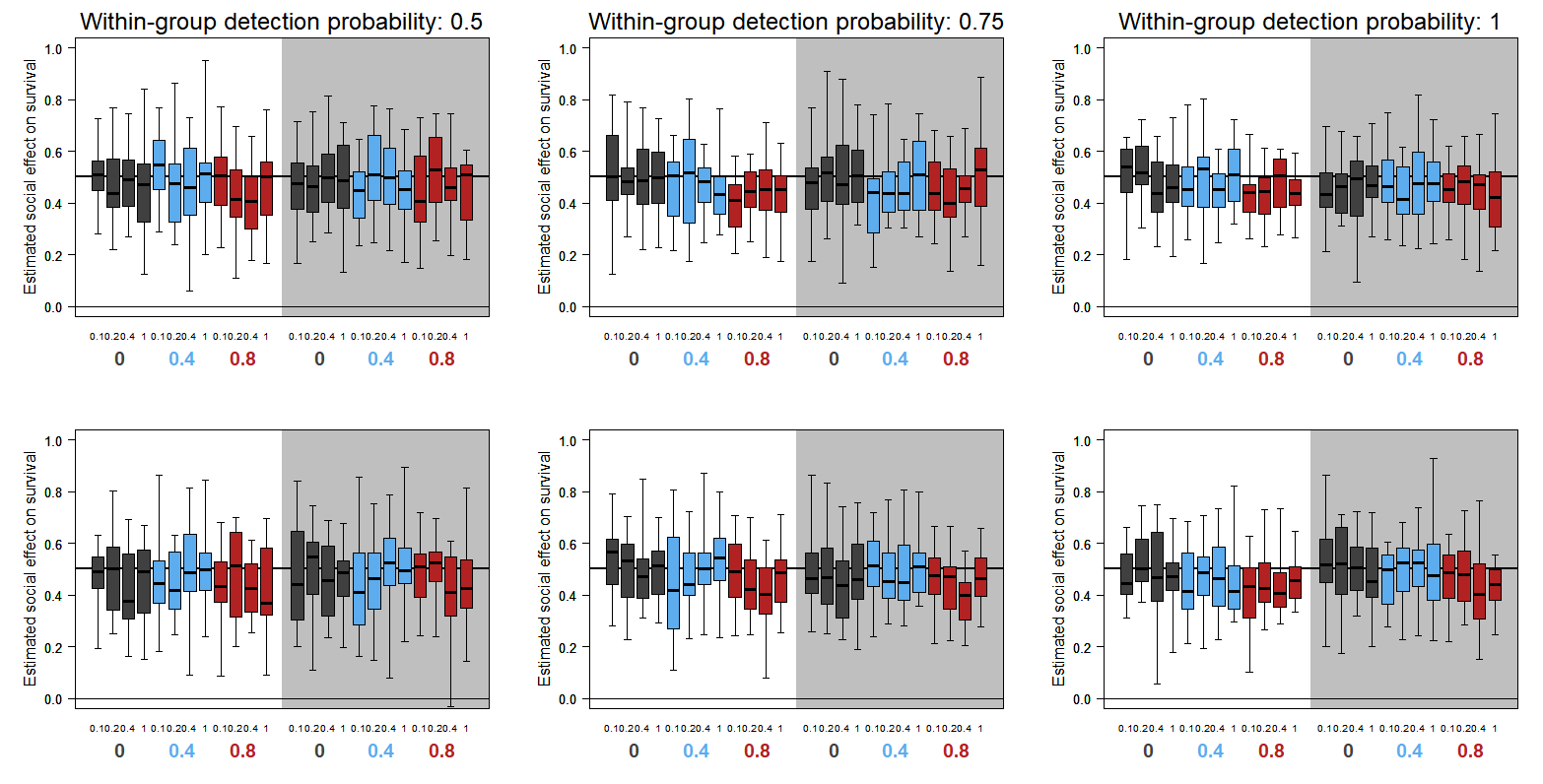


Figure S11. The impacts of sampling design (within-plot: sets of boxes of the same colour), within-group detection probability (columns) and social structure (rows) on Cormack-Jolly-Seber estimates for the sex effect on survival probability for a range of simulated effect sizes (colours of boxes). Boxplots show the distribution of posterior medians from multiple simulation runs with the solid line the median, boxes the interquartile range and whiskers the full range of values. We illustrate contexts in which a local measure of centrality (strength) and global measure of centrality (betweenness) are used as explanatory variables. The black line indicates the value used for simulations.


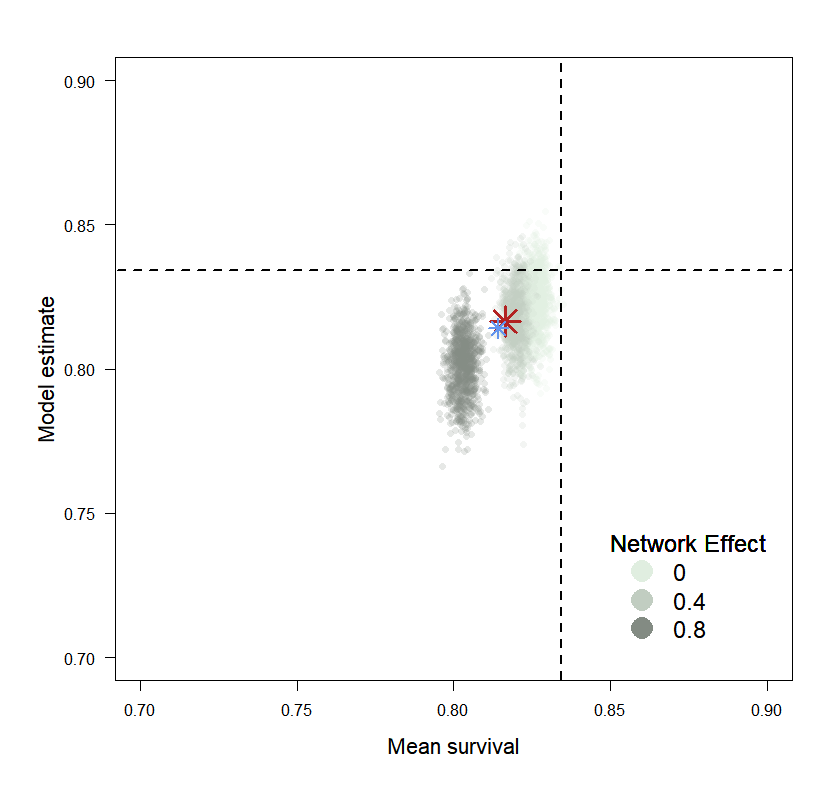


Figure S12. Plot showing mean survival probabilities in the simulations versus model estimated survival probabilities (assuming equal numbers of each sex) from each simulation run. Dotted lines indicate the expected mean based on simulation inputs assuming equal numbers of each sex. The strength of the network effects is indicated by the colour of the points. While model estimates accurately reflect the mean values of survival generated by the simulations, these are downward-biased from input values when there are stronger network effects on survival.
