# Supplementary material for: Generation and applications of simulated datasets to integrate social network and demographic analyses
Source: Ecol Evol. 2023 May 15;13(5):e9871. doi: 10.1002/ece3.9871 (PMC10185435; doi:10.1002/ece3.9871)
Supplement: Supplementary file 2 — Appendix S2 [file ECE3-13-e9871-s002.docx]

**Generation and applications of simulated datasets to integrate social network and demographic analyses: Supplementary Materials 2**

Matthew J Silk^1^* and Olivier Gimenez^1^

1 CEFE, Univ Montpellier, CNRS, EPHE, IRD, Montpellier, France

*corresponding author: [matthewsilk@outlook.com](mailto:matthewsilk@outlook.com)

**genNetDem Key functions**

*Population features*

The **population_generation_basic()** function generates basic data for a group-structured population distributed uniformly in 2D space. The function takes two arguments: *n* defines the population size and *ng* the number of groups in the population. When *n*=*ng* individuals are distributed uniformly across the defined coordinates. When *n*>*ng* groups are distributed uniformly across the same coordinates with individuals in the same group sharing the same spatial location. Groups are generated stochastically meaning that groups will be similar but not identical in size. The function returns a dataframe including information on group membership and spatial location together with a distance matrix for all individuals in the population.

The **indiv_info_gen()** and **indiv_info_add()** functions provide flexibility in generating and updating individual-level trait data respectively. The indiv_info_gen() function uses the indiv_data dataframe and either an existing version of itself or a simple vector of individual IDs as its main inputs. Variables can be specified as covariates or categorical factors, with further arguments specifying additional features of the variable. For example, it is possible to specify the distribution of a covariate (uniform, normal or log-normal) or the number of levels and level names of a factor. For factors the prob_levels argument can also be used to control the proportion of individuals in the population assigned to each level of the factor. The output is a list containing a dataframe of individual traits and information on the variables. The latter is stored alongside the dataframe to make it easier to update when individuals enter and/or leave the population Updating uses the indiv_info_add() function which simply requires the indiv_info dataframe and information on the variables generated from indiv_info_gen() alongside the indiv_data dataframe as inputs and returns the same list as indiv_info_gen().

The **timestep_demographics()** function controls survival and recruitment in the simulated population. Survival is stochastic based on each individual’s survival probability. The recruitment rate is calculated from population mean survival probability and implemented stochastically via a draw from a Poisson distribution. Individuals are recruited into groups. When *n*=*ng* (each individual is its own group) individuals are recruited into empty group locations to replace dead individuals where possible and new locations when more individuals are recruited than die. When *n*>*ng* (i.e. true groups are present in the population) then individuals are more likely to be sampled into smaller groups. The function returns a) a newly updated indiv_data dataframe, b) a new distance matrix, and c) updates the full_indiv_data dataframe that combines all individuals ever present in the population.

*Survival features*

The **covariates_survival()** function calculates survival probabilities for each individual. Effects of individual traits are simulated using output from the indiv_info_gen() function, with both covariates and categorical factors allowed. Effect sizes are specified as a list with each element corresponding to a different trait (scalar for covariates and a vector corresponding to each factor levels for categorical variables). There is considerable flexibility in which measures of social network position can be included as covariates; both the function and R package used can be specified within the function, with additional functionality for most common packages such as sna, igraph and tnet provided for particular measures (e.g. code that extracts the right part of the object calculated for each metric calculation, by inverting edge weights to calculate betweenness and closeness centrality in igraph, etc.). Effect sizes are provided in the same way as for non-network covariates. It is also possible to simulate network covariance in survival whereby closely connected individuals have either more or less similar survival probabilities than expected by chance. Network covariance is simulated by using an approximation of the network as a covariance matrix. This approximation step is required to ensure that the matrix is positive definite. It is conducted using nearPD() function from the Matrix package. The covMat_check argument can be set to TRUE if a user wants to check the correlation of the resulting covariance matrix with the inputted social network. The output of the function is the indiv_data dataframe with updated survival probabilities. Currently, covariates_survival() simulates independent (additive) effects of traits, meaning there is currently no functionality to capture interactions among variables (e.g. network position having different effects in males than females).

There is also a simpler **basic_survival()** function that generates survival probabilities with a normal distribution on the logit scale without any functionality to include covariates.

*Network features*

The **network_generation_covariates()** function provides a sophisticated way of generating underlying network structure (Fig. S12). Key data inputs to the function include the indiv_data and indiv_info dataframes and the distance matrix for the population. Current functionality is focussed on how these traits may impact the probability of forming social connections within and between groups separately thus employing the stochastic block model as a generative model (see main text for references). Edge probabilities and edge weights are modelled independently to allow variables to explain variation in one or both of them. Edge weights are parameterised by fitting a beta distribution to a provided mean and variance, generating edge weights between 0 and 1 in the underlying network. Increasing the d_effp and d_effw arguments increases the importance of the distance between groups in the probability that an edge exists and the weight of edges that do exist respectively. They are both implemented by multiplying baseline values by ${\frac{1}{d\_eff}}^{distance}$. Covariate effects on network properties are implemented using the covs and effs arguments. The covs argument indicates the columns of the indiv_info dataframe that impact network structure. The effs argument is a list of effect sizes for each factor that has an effect; each element is either a vector of eight values (for covariates) corresponding to the p_ig, wi_m, wi_v, p_og, wo_m, wo_v, d_effp and d_effw parameters or an eight-by-number of factor levels matrix (for factors). Setting plot=TRUE will provide a basic visualisation of the network. The function returns the network as an adjacency matrix and igraph object.


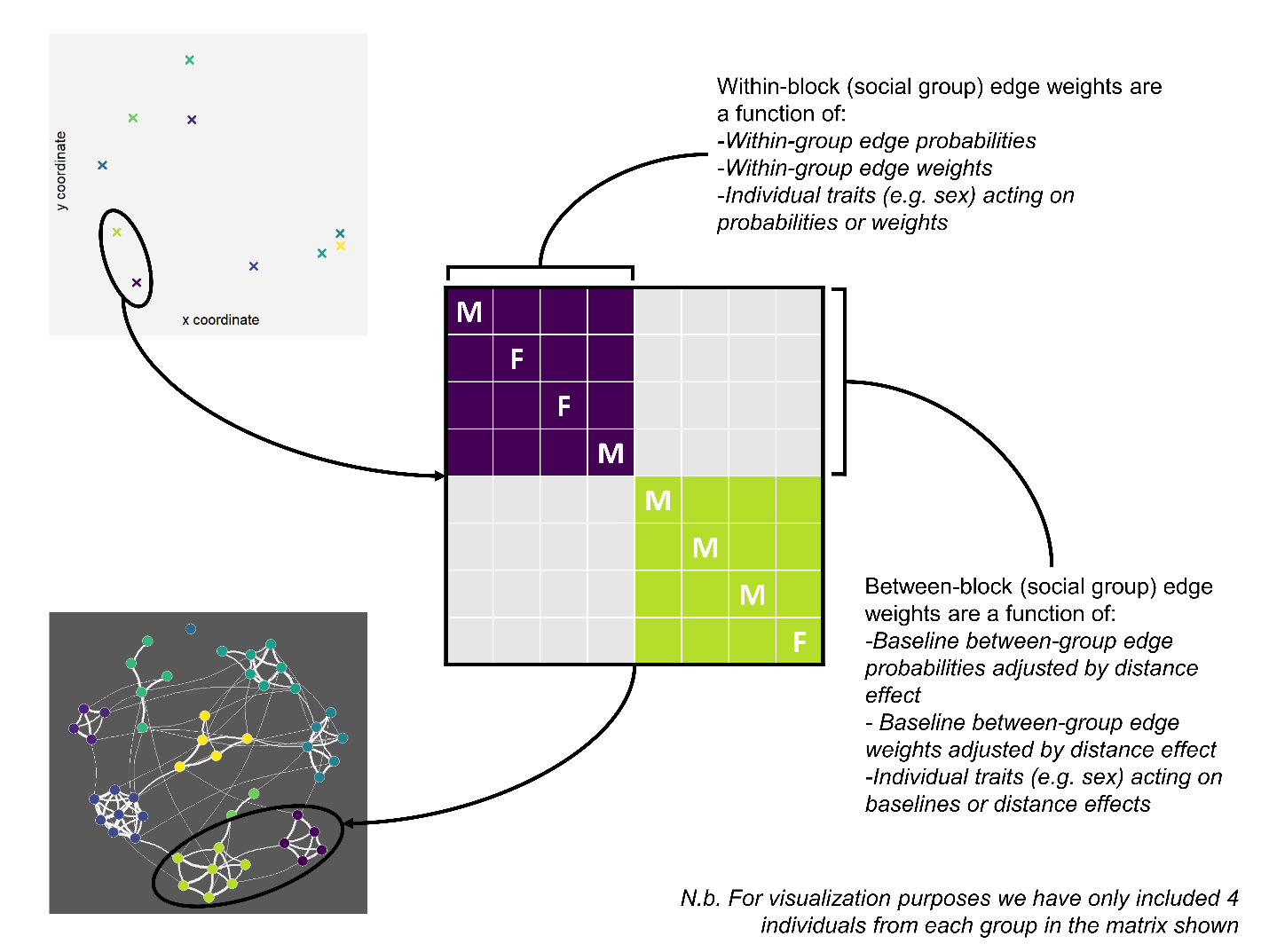


*Figure S13. An illustration of the generative model used to simulate underlying dyadic social networks in network_generation_covariates().*

There is also a simpler **network_generation_basic()** function that uses the same spatial/block generative model but does not include covariates. For this function the beta distribution for edge weights is parameterised directly rather than with its mean and variance. This is less intuitive but also more robust to user input.

The **network_rewire_covariates()** function provides a way to add newly recruited and remove dead individuals from the underlying network. The arguments are largely identical to those for network_generation_covariates(), although the existing network is also required as an input. It also provides functionality to select probabilities that a) an individual changes some of its social relationships (p_wr_i) and b) each social relationship for selected individuals changes (p_wr_e). This allows considerable flexibility in the stability in the network over time. It is also possible to change the parameters used to generate the network if desired.

There are two main functions that generate grouping events based on the network: **interaction_generation_simul()** and **interaction_generation_seq()**. The former divides all individuals in the population into interactions (or isolates) at any one point in time while the latter independently samples one grouping event/interaction of a defined size from the population at a time. The former is more widely useful and so is described in greater detail. Key inputs are data on individual IDs, their underlying social network (as an adjacency matrix) and a mean group size to divide the populations into groups, with group membership being stored in a group-by-individual matrix (GBI). The n_ts argument defines the number of times this process should be repeated (the number of “behavioural timesteps”). Assigning individuals into groups based on the underlying network can create computational challenges if unconstrained. We use a similar approach to Evans et al. (2020), with individuals being added to groups sequentially and the probability of an individual joining a group being proportional to the strength of its social relationships with existing group members. The pm, pow and float arguments all adjust how these probabilities are defined. The probability of adding an individual to an interaction is proportional to: the product of ${(product of edge weights+ \frac{sum of edge weights}{pm}+float)}^{pow}$ for all individual already selected into the group. The function returns the full GBI and a vector indicating which groups were observed in each behavioural timestep. The interaction_generation_seq() function is similar but draws a pre-specified number of groups independently from the population. This is computationally less challenging and may be accurate for some sampling strategies but is less effective for generating a true set of grouping events.

Finally two **network_checker()** functions can be used to quantify and visualise how well the social network derived from simulated grouping events matches the underlying social network these grouping events were generated with. By default the **network_checker_seq()** function will: 1) plot the network generated from grouping event data; 2) plot relationships between a series of common centrality measures in the underlying network (degree, strength, betweenness and closeness) and the number of grouping events an individual is observed in (using interaction_generation_seq() function will typically result in a positive correlation between centrality and gregariousness); 3) conduct a basic matrix regression using the netlm function in the R package sna (using qapspp as the null hypothesis permutation test) to test how edge weights in the underlying network and those in the network generated from grouping events are related; and 4) plot relationships between commonly-used centrality measures calculated from the two different networks (again using degree, strength, betweenness and closeness). Users can also provide additional arguments to the function compare other individual-level measures between the underlying network and the one generated from grouping event data. The **network_checker_simul()** performs in the same way for interaction_generation_simul() function, sharing the same functionality aside from output 2 – as all individuals occur in the same number of grouping events.

*Observation features*

Two main observation features are provided. There are two cap_and_obs() functions that generate an observed network dataset based on information provided to the function about sampling strategy and design. The cap_dat_gen() function transforms these network datasets into typical capture histories for use in capture-recapture analyses. It outputs capture histories for both behavioural timesteps and demographic timesteps (see Workflow section).

The functions **cap_and_obs()** and **cap_and_obs2()** provide considerable flexibility in how grouping events are sampled. Of the two versions **cap_and_obs2()** has slightly wider functionality. The inputs to both versions include: a) data on true grouping events (the GBI and a vector indicating which behavioural timestep each group occurred in); b) an indication of the behavioural timesteps to be sampled, separately for captures and observations; c) the success of sampling including both the proportion of groups observed/captured by the sampler and the proportion of individuals in each sampled group that are observed or captured; and d) a vector which (if any) individuals had been captured previously. There are two key differences between cap_and_obs() and cap_and_obs2(). Firstly, in the former a minimum behavioural timestep, maximum behavioural timestep and interval are provided separately for capture and observation windows and the sequence of captures and observations are calculated internally within the function. In contrast the inputs for the latter are vectors indicating the behavioural timesteps in which captures and observations will take place, providing greater flexibility for irregular sampling designs. Secondly, cap_and_obs() has a single pci input specifying the probability of an individual being detected in both captured and observed groups, while cap_and_obs2() has separate pci and pmi arguments allowing these two probabilities to be specified independently. Both functions return a list containing the full GBI but for only captured groups, a vector indicating which groups were captured, the full GBI for observed and captured groups combined and a vector indicating which groups were observed. An **obs_net_checker()** function can be used to compare the properties of the sampled network to both the dyadic social network calculated from grouping event data and the underlying social network of the population. It works much the same way as the other network_checker functions, including exploiting the netlm function in sna and the ability to provide additional user-specified social network measures if preferred.

The **cap_dat_gen()** function is a convenience function that can be used to transform the outputs of common workflows for genNetDem() to conventional capture history datasets for demographic modelling. Inputs provided are a) CG - the list of observed GBIs; b) SW_store - a list of all sampling windows as output from interaction_generation_simul(); c) full_indiv_dat - the full individuals dataset containing information on the ID, group and spatial behaviour of all individuals; d) inds_alive - a list of individuals recorded alive at each demographic timestep; and e) bs - the number of behavioural timesteps per demographic timestep. It returns capture histories for both behavioural and demographic timesteps as a list.

**References**

Evans, J., Fisher, D.N., & Silk, M.J. (2020). The performance of permutations and exponential random graph models when analyzing animal networks. *Behavioral Ecology*, 31(5), 1266-1276.
